# Supplementary material for: Prevalence of homebirth preference and associated factors among pregnant women in Ethiopia: Systematic review and meta-analysis
Source: PLoS One. 2023 Nov 15;18(11):e0291394. doi: 10.1371/journal.pone.0291394 (PMC10651027; doi:10.1371/journal.pone.0291394)
Supplement: Attachment — Submitted filename: Revised-PONE-D-23-02877.pdf [file pone.0291394.s004.pdf]

PLOS ONE

# PREVALENCE OF HOMEBIRTH PREFERENCE AND ASSOCIATED FACTORSAMONG PREGNANT WOMEN IN ETHIOPIA: SYSTEMATIC REVIEW AND META-ANALYSIS

--Manuscript Draft--

| | |
|---|---|
| **Manuscript Number:** | PONE-D-23-02877 |
| **Article Type:** | Research Article |
| **Full Title:** | PREVALENCE OF HOMEBIRTH PREFERENCE AND ASSOCIATED FACTORSAMONG PREGNANT WOMEN IN ETHIOPIA: SYSTEMATIC REVIEW AND META-ANALYSIS |
| **Short Title:** | PREVALENCE OF HOMEBIRTH PREFERENCE AND ASSOCIATED FACTORS |
| **Corresponding Author:** | Jira Wakoya Feyisa, MPH<br>Wollega university<br>Nekemte, Oromia ETHIOPIA |
| **Keywords:** | Homebirth preference, pregnant women, systematic review, Ethiopia |
| **Abstract:** | Background: The homebirth preference is the intention/plan of pregnant women to give birth at their own homes. Planned births outside of health care facilities and a desire for homebirth lead to care-seeking delays and are connected to multiple stillbirths and postpartum morbidities. Results from some studies were inconsistent and inconclusive. Therefore, this study aimed to estimate the pooled prevalence of homebirth preference and associated factors among pregnant women in Ethiopia.<br>Methods: The research articles used for this systematic review and meta-analysis were identified through Google Scholar, Medline, Pub Med, Cochrane library, and the Web of Science search engines from 20th August 2022 to 6th November 2022. A standardized data extraction checklist and Stata version 14 for analysis were used. Heterogeneity was assessed by the Cochrane Q test statistic and I2 statistics. To estimate the pooled prevalence of homebirth preference, a random-effects model was fitted. Association between homebirth preference and independent variables was reported using an odds ratio with a 95% confidence interval. Publication bias was checked visually using a funnel plot and Egger's test was used to check the significant presence of publication bias.<br>Results: A total of 976 research articles were identified from several databases and seven studies satisfied eligibility criteria and were included in the systematic review and meta-analysis. The pooled prevalence of homebirth preference in Ethiopia was 39.62% (95% CI 27.98, 51.26). The current meta-analysis revealed that average monthly income <1800 ETB (OR = 2.66, 95% CI 1.44, 4.90) lack of antenatal care follow-up (OR = 2.57, 95%CI 1.32, 5.01), being multipara (OR = 1.77, 95%CI 1.39, 2.25), poor knowledge about obstetric danger sign (OR = 5.75, 95%CI 1.o2, 32.42), and not discussing the place of delivery with a partner (OR = 5.89 (95%CI 1.1, 31.63) were significantly associated with homebirth preference.<br>Conclusion: This systematic review and meta-analysis examined the high prevalence of homebirth preference. This high prevalence of homebirth preference was associated with low average monthly income (<1800 ETB), lack of ANC follow-up, multipara, poor knowledge about obstetric danger signs, and not discussing with their partner the place of delivery. Improving the knowledge of pregnant women about obstetric danger signs is necessary to decrease the prevalence of homebirth preference. |
| **Order of Authors:** | Jira Wakoya Feyisa, MPH |
| | Emiru Merdassa |
| | Matiyos Lema, MPH |
| | Wase Benti Hailu, MPH |
| | Markos Desalegn |
| | Adisu Tafari Shama |
| | Debela Dereje Jaleta |

Powered by Editorial Manager® and ProduXion Manager® from Aries Systems Corporation

| | |
|---|---|
| | Gamachis Firdisa Tolasa, Msc |
| | Robera Demissie Berhanu, Msc |
| | Solomon Seyife Alemu |
| | Keno Melkamu Kitila |
| **Additional Information:** | |
| **Question** | **Response** |
| **Financial Disclosure**<br><br>Enter a financial disclosure statement that describes the sources of funding for the work included in this submission. Review the submission guidelines for detailed requirements. View published research articles from *PLOS ONE* for specific examples.<br><br>This statement is required for submission and **will appear in the published article** if the submission is accepted. Please make sure it is accurate.<br><br>**Unfunded studies**<br>Enter: *The author(s) received no specific funding for this work.*<br><br>**Funded studies**<br>Enter a statement with the following details:<br>• Initials of the authors who received each award<br>• Grant numbers awarded to each author<br>• The full name of each funder<br>• URL of each funder website<br>• Did the sponsors or funders play any role in the study design, data collection and analysis, decision to publish, or preparation of the manuscript?<br>• **NO** - Include this sentence at the end of your statement: *The funders had no role in study design, data collection and analysis, decision to publish, or preparation of the manuscript.*<br>• **YES** - Specify the role(s) played.<br><br>* typeset | The authors received no specific funding for this work |
| **Competing Interests**<br><br>Use the instructions below to enter a competing interest statement for this submission. On behalf of all authors, disclose any competing interests that | The authors have declared that no competing interests exist |

| | |
|---|---|
| could be perceived to bias this work—acknowledging all financial support and any other relevant financial or non-financial competing interests.<br><br>This statement is required for submission and **will appear in the published article** if the submission is accepted. Please make sure it is accurate and that any funding sources listed in your Funding Information later in the submission form are also declared in your Financial Disclosure statement.<br><br>View published research articles from *PLOS ONE* for specific examples.<br><br>**NO authors have competing interests**<br><br>Enter: *The authors have declared that no competing interests exist.*<br><br>**Authors with competing interests**<br><br>Enter competing interest details beginning with this statement:<br><br>*I have read the journal's policy and the authors of this manuscript have the following competing interests: [insert competing interests here]*<br><br>* typeset | |
| **Ethics Statement**<br><br>Enter an ethics statement for this submission. This statement is required if the study involved:<br><br>• Human participants<br>• Human specimens or tissue<br>• Vertebrate animals or cephalopods<br>• Vertebrate embryos or tissues<br>• Field research<br><br>Write "N/A" if the submission does not require an ethics statement. | N/A |

| | |
|---|---|
| General guidance is provided below. Consult the submission guidelines for detailed instructions. **Make sure that all information entered here is included in the Methods section of the manuscript.**<br><br>**Format for specific study types**<br><br>**Human Subject Research (involving human participants and/or tissue)**<br>• Give the name of the institutional review board or ethics committee that approved the study<br>• Include the approval number and/or a statement indicating approval of this research<br>• Indicate the form of consent obtained (written/oral) or the reason that consent was not obtained (e.g. the data were analyzed anonymously)<br><br>**Animal Research (involving vertebrate animals, embryos or tissues)**<br>• Provide the name of the Institutional Animal Care and Use Committee (IACUC) or other relevant ethics board that reviewed the study protocol, and indicate whether they approved this research or granted a formal waiver of ethical approval<br>• Include an approval number if one was obtained<br>• If the study involved *non-human primates*, add *additional details* about animal welfare and steps taken to ameliorate suffering<br>• If anesthesia, euthanasia, or any kind of animal sacrifice is part of the study, include briefly which substances and/or methods were applied<br><br>**Field Research**<br><br>Include the following details if this study involves the collection of plant, animal, or other materials from a natural setting:<br>• Field permit number<br>• Name of the institution or relevant body that granted permission | |
| **Data Availability**<br><br>Authors are required to make all data | Yes - all data are fully available without restriction |

| | |
|---|---|
| underlying the findings described fully available, without restriction, and from the time of publication. PLOS allows rare exceptions to address legal and ethical concerns. See the PLOS Data Policy and FAQ for detailed information.<br><br>A Data Availability Statement describing where the data can be found is required at submission. Your answers to this question constitute the Data Availability Statement and **will be published in the article**, if accepted.<br><br>**Important:** Stating 'data available on request from the author' is not sufficient. If your data are only available upon request, select 'No' for the first question and explain your exceptional situation in the text box.<br><br>Do the authors confirm that all data underlying the findings described in their manuscript are fully available without restriction? | |
| **Describe where the data may be found in full sentences. If you are copying our sample text, replace any instances of XXX with the appropriate details.**<br><br>• If the data are **held or will be held in a public repository**, include URLs, accession numbers or DOIs. If this information will only be available after acceptance, indicate this by ticking the box below. For example: *All XXX files are available from the XXX database (accession number(s) XXX, XXX.*).<br>• If the data are all contained **within the manuscript and/or Supporting Information files**, enter the following: *All relevant data are within the manuscript and its Supporting Information files.*<br>• If neither of these applies but you are able to provide **details of access elsewhere**, with or without limitations, please do so. For example:<br><br>*Data cannot be shared publicly because of [XXX]. Data are available from the* | all data are fully available |

| | |
|---|---|
| *XXX Institutional Data Access / Ethics Committee (contact via XXX) for researchers who meet the criteria for access to confidential data.*<br><br>*The data underlying the results presented in the study are available from (include the name of the third party and contact information or URL).*<br>• This text is appropriate if the data are owned by a third party and authors do not have permission to share the data.<br><br>* typeset | |
| Additional data availability information: | Tick here if the URLs/accession numbers/DOIs will be available only after acceptance of the manuscript for publication so that we can ensure their inclusion before publication. |

Manuscript

Click here to access/download;Manuscript;systematic review and meta-analysis.docx

# PREVALENCE OF HOMEBIRTH PREFERENCE AND ASSOCIATED FACTORS AMONG PREGNANT WOMEN IN ETHIOPIA: SYSTEMATIC REVIEW AND META-ANALYSIS

[1*]Jira Wakoya Feyisa, [1]Emiru Merdassa, [1]Matiyos Lema, [1]Wase Benti Hailu, [1]Markos Desalegn, [1]Adisu Tafari Shama, [2] Debela Dereje Jaleta, [2]Gamachis Firdisa Tolasa, [3]Robera Demissie Berhanu, [4]Solomon Seyife Alemu [5]Keno Melkamu Kitila

Email addresses: jirawakoya462@gmail.com (J. Wakoya), emiruydm2016@yahoo.com (E. Merdassa), maatii3399@gmail.com (M. Lema), wasben333@gmail.com (W. Benti), markosdesalegn@gmail.com (M. Desalegn), adisuteferi1906@gmail.com (A. Tafari), debeladereje4633@gmail.com (D. Dereje), gamachis.firdisa@gmail.com (G. Firdisa), roobeeraadb@gmail.com (R. Demissie), SoleSeifa@gmail.com (S. Seyife) qannoo63@gmail.com (K. Melkamu)

***Correspondent Author**: Jira Wakoya Feyisa, E-mail: jirawakoya462@gmail.com,

Phone: +251-923-00-95-83/+251-910-80-53-50, P.O.BOX: 395, Nekemte, Ethiopia

## ABSTRACT

**Background:** The homebirth preference is the intention/plan of pregnant women to give birth at their own homes. Planned births outside of health care facilities and a desire for homebirth lead to care-seeking delays and are connected to multiple stillbirths and postpartum morbidities. Results from some studies were inconsistent and inconclusive. Therefore, this study aimed to estimate the pooled prevalence of homebirth preference and associated factors among pregnant women in Ethiopia.

**Methods:** The research articles used for this systematic review and meta-analysis were identified through Google Scholar, Medline, Pub Med, Cochrane library, and the Web of Science search engines from 20th August 2022 to 6th November 2022. A standardized data extraction checklist and Stata version 14 for analysis were used. Heterogeneity was assessed by the Cochrane Q test statistic and I2 statistics. To estimate the pooled prevalence of homebirth preference, a random-effects model was fitted. Association between homebirth preference and independent variables was reported using an odds ratio with a 95% confidence interval. Publication bias was checked visually using a funnel plot and Egger's test was used to check the significant presence of publication bias.

**Results:** A total of 976 research articles were identified from several databases and seven studies satisfied eligibility criteria and were included in the systematic review and meta-analysis. The pooled prevalence of homebirth preference in Ethiopia was 39.62% (95% CI 27.98, 51.26). The current meta-analysis revealed that average monthly income <1800 ETB (OR = 2.66, 95% CI 1.44, 4.90) lack of antenatal care follow-up (OR = 2.57, 95%CI 1.32, 5.01), being multipara (OR = 1.77, 95%CI 1.39, 2.25), poor knowledge about obstetric danger sign (OR = 5.75, 95%CI 1.o2,

32.42), and not discussing the place of delivery with a partner (OR = 5.89 (95%CI 1.1, 31.63) were significantly associated with homebirth preference.

**Conclusion**: This systematic review and meta-analysis examined the high prevalence of homebirth preference. This high prevalence of homebirth preference was associated with low average monthly income (<1800 ETB), lack of ANC follow-up, multipara, poor knowledge about obstetric danger signs, and not discussing with their partner the place of delivery. Improving the knowledge of pregnant women about obstetric danger signs is necessary to decrease the prevalence of homebirth preference

**Keywords:** Homebirth preference, pregnant women, systematic review, Ethiopia

## BACKGROUND

Homebirth preference is the intention/plan of pregnant women to give birth at their own homes [1]. Planned births outside of health care facilities and a desire for homebirth lead to care-seeking delays and are probably connected to multiple stillbirths and postpartum morbidities [2]. Once the mother planned to give birth at home the probability of giving birth in healthcare facilities is reduced which increases the risk of maternal fatality rate but in Sub-Saharan Africa, many women still prefer to give birth at home [3]. Even though Ethiopia has devised Critical strategies including promoting institutional delivery services for lowering maternal morbidity and mortality, just 1 in 4 women gave birth in a medical facility in the country [4].

In Ethiopia, a substantial percentage of pregnant mothers prefer to give birth at home [5]. According to the 2019 Mini Ethiopian Demographic and Health Survey report, of all live births in the five years before the survey, only 50% were delivered by a skilled provider [6]. In the developing regions of Ethiopia, more than three-fourths of mothers gave birth at home [4].

Although most maternal health care is free in Ethiopia, home birth rates are reported to be high [7]. In mothers who gave birth at home, maternal factors accounted for nearly half of infant deaths, premature birth, umbilical cord infection, being accidentally knocked on the head during delivery, breach presentation, and severe maternal anemia are the main causes of infant mortality [8]. Patients with the postpartum hemorrhage who gave birth outside of a hospital had a higher risk of receiving blood transfusion [9].

In Ethiopia, different studies have been conducted to determine the homebirth preference and associated factors among pregnant women which show a great variation across different geographical areas and periods due to different factors such as lack of transportation, low awareness of the advantages of skilled attendance at delivery, little role in making decisions and economic constraints and not discussing the place of delivery with a partner [1, 4, 5, 10].

To guarantee the prevention, detection, and management of problems, all women should have access to skilled care during pregnancy and childbirth. Despite Ethiopia having created several measures by promoting institutional delivery services, home deliveries are still widespread, especially in remote areas [1, 5].

Even though studies have reported on homebirth preference and associated factors among pregnant women in Ethiopia, there is no nationally representative pooled data on homebirth preference and associated factors among pregnant women in Ethiopia. Furthermore, previous studies' report varies between regions within the country. Therefore, this systematic review and meta-analysis aimed to provide more current and representative national data on homebirth Preference and associated factors in Ethiopia.

**Methods**

**Search strategy**

From the very beginning, the PROSPERO database and database of abstracts of reviews of effects (DARE) (http://www.library.UCSF.edu) were searched to check whether published and/or ongoing projects exist related to the topic. The research articles search strategy, selection of studies, data extraction, and result reporting were done in accordance with Preferred Reporting Items for Systematic Reviews and Meta-Analyses (PRISMA) guidelines [11]. A PICO principle was adapted for searching terms. (PRISMA) guidelines were used to state this systematic review and meta-analysis [12]. The research articles used for this systematic review and meta-analysis were identified through Google Scholar, Medline/Pub Med, Cochrane library, the Web of Science, Hinari, Science Direct, WoS, ProQuest, African Journals Online, and online university repositories (University of Gondar, Addis Ababa, Jimma and Haramaya University) search engines by developing search strategies. Boolean operators such as OR, AND, and NOT were used with search terms such as "Preference" "Desire" "need" "intention" "choice "homebirth" "home delivery" "Place of delivery" "place of Birth" "unskilled birth attendant" "associated factors" "determinants" "risk factors" "predictors") "reproductive age women" "pregnant women/mother" "Ethiopia". Identified research articles were screened to make sure that all relevant literature was included. Literature was downloaded to Endnote (version X7.8) to maintain and manage citations, and facilitate the review process [13].

**Eligibility criteria**

In this systematic review and meta-analysis, we included all studies that were conducted on homebirth preference and associated factors among pregnant women in Ethiopia. The participants were pregnant women/mothers. We included all types of articles that were published in the form of journal articles, master's theses, and dissertations in the English language.

Published studies were included in the study, but unpublished studies were not found. Full research articles, which were not accessed after at least two email contacts of the primary author, were not included because of the failure to assess the quality of articles in the absence of full text. The restriction was made for the date of publication, articles published before 2016 were excluded because the Ethiopian strategies to overcome the problem of homebirth were critically started since 2016 as transformation strategies. All studies conducted in Ethiopia were included.

**Outcome measurement**

There were two main outcomes. The first outcome of interest was the prevalence, which was estimated as the total number of women preferring homebirth, cases divided by the total number of sample sizes multiplied by 100. From the primary study, homebirth preference was operationalized as the choice or desire, or intention of women to give birth at their own homes. The second outcome was the factors associated with homebirth preference, which was determined using the odds ratio (OR) and calculated based on binary outcomes from the included primary studies. The variables included in the review were education, average monthly income, transport access, ANC follow-up (Yes versus No), parity (primipara versus multipara), previous place of delivery (home/health institution), discussion on a place of delivery with a partner (discussed/not discussed) and knowledge (poor/ knowledgeable). Women were asked about a history of ANC follow-up for the current pregnancy. The variable was measured by binary variable "Yes" for those women who used one and above ANC visit and "No" for women who did not visit ANC during the current pregnancy.

**Data collection and quality assessment**

To assess the quality of the included study, the Joanna Briggs Institute (JBI) quality appraisal checklist for cross-sectional studies quality assessment tool was used [14]. Three data extractors (JW, EM and ML) extracted data by using a standardized data extraction checklist on Microsoft Excel. Reference management software (Endnote version X7.8) was used to combine search results from databases and to remove duplicate articles initially. Then, research articles were screened and excluded by their abstracts and titles. Full-text articles or reports were assessed for the remaining research articles. Based on the preset inclusion and exclusion criteria, the eligibility of the primary studies was evaluated. For the first outcome (prevalence of homebirth preference), the data extraction checklist included the authors' name, year of publication, region (an area where studies were conducted), study design, sample size, response rate, and the number of participants with the homebirth preference. For the second outcome (factors associated with homebirth preference), data were extracted in two by two tables format, and then the log OR was computed based on the findings of the primary articles. Inconsistencies between three independent reviewers were fixed by including other reviewers (MD, AT, WB and KM) after discussion for possible agreement. Corresponding authors of the research articles were contacted through their email when the included primary articles did not have sufficient data.

**Data analysis and synthesis**

Using a format prepared in a Microsoft Excel spreadsheet, necessary information from each original study was extracted. Then the data were exported to STATA version 14.0 and used to calculate the pooled effect size with 95% CI. To check heterogeneity among the included studies, the Cochran Q test (Chi-squared statistic) and I2 statistic on forest plots were computed. Cochran’s Q statistical heterogeneity test is declared statistically significant at $p \leq 0.05$. I2 statistics range from 0 to 100% and I2 statistic values of 0, 25, 50, and 75% were considered as

no, low, moderate, and high degrees of heterogeneity, respectively [15]. A random-effects model was used to determine the pooled prevalence of homebirth preference and the pooled effect size of associated factors when a high degree of heterogeneity was observed for the first and second outcomes. To identify the source of potential random variation subgroup analysis was conducted based on the region where the studies were conducted and based on the timing of the study. Meta-regression was computed to see the presence of statistically significant heterogeneity. Publication bias was assessed by using a funnel plot. The symmetry of the funnel plot is an indicator of the absence of publication bias (fig 5). In addition, Egger's (p-value= 0.079 weighted regression and Begg's tests (p-value= 0.051) were used to check the absence of publication bias. Statistical nonsignificance of publication bias was declared at a p-value of greater than 0.05 [16].

**Result**

A total of 976 studies were identified from various electronic databases and library catalogs. Of these studies, 646 articles records were identified and removed due to duplication. Reviewing titles and abstracts resulted in the exclusion of 303 irrelevant research articles for our study. After assessing the full texts of the remaining articles, 20 studies were excluded as they did not meet the preset eligibility criteria. The remaining seven (7) studies were included in the final systematic review and meta-analysis (Fig. 1).

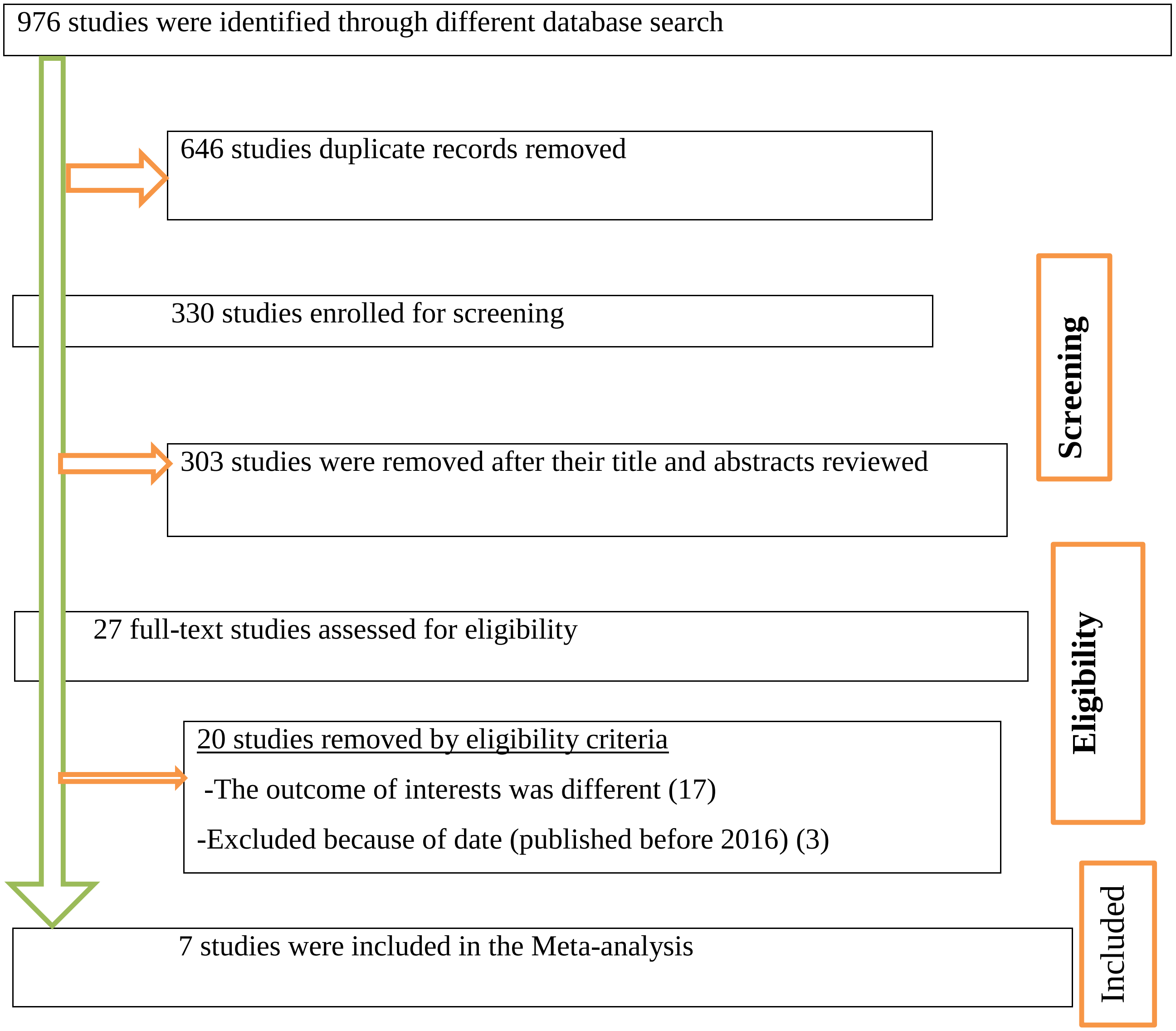


Fig.1 PRISMA flow diagram of included studies in the systematic review and meta-analysis of the prevalence of homebirth preference and associated factors among pregnant women in Ethiopia, 2022

**Characteristics of included studies**

All of the seven studies included in this study were published between 2019 to 2022 in peer review journals [5, 10, 17-21]. A total of 3458 study participants were included in the current systematic review and meta-analysis. The smallest sample size was 346 from a study conducted in the Amhara region, Simada district [18], and the largest sample size was 769 a study conducted in SNNPR [10]. All included studies were cross-sectional in study design with 4

prospective [5, 17, 19, 21] and 3 retrospective cross-sectional study designs [10, 18, 20]. Regarding study setting, two studies were conducted in the Amhara region [18, 19], two studies in the SNNPR [10, 21], one study was from the Afar region [17], one study was conducted in the Oromia region [20] and one study conducted from PMA survey data [5]. The summary of the included articles is described in (Table 1).

Table.1 Summary of Included Studies on the prevalence of homebirth preference in Ethiopia

| SN | Author | Publication year | Region | Study area | Study design | Sample size | Quali score | Prevalence(95%CI) |
|---|---|---|---|---|---|---|---|---|
| 1 | Alemu et al [10] | 2019 | SNNR | Wonago | Cross-sectional | 769 | 7 | 25.62(22.53,28.70) |
| 2 | Kahsay et al [17] | 2019 | Afar | Zone 3 | Cross-sectional | 357 | 7 | 69.75(64.98,74.51) |
| 3 | Mekie et al [18] | 2019 | Amhara | Simada | Cross-sectional | 346 | 8 | 56.36(51.13,61.58) |
| 4 | Tsegaye et al [19] | 2019 | Amhara | Debretabor | Cross-sectional | 394 | 7 | 29.19(24.70,33.68) |
| 5 | Bekuma et al [20] | 2020 | Oromia | Jimma Arjo | Cross-sectional | 506 | 8 | 39.53(35.27,43.79) |
| 6 | Alemu et al [21] | 2022 | SNNPR | Arba Minch S.S | Cross-sectional | 408 | 9 | 24.02(19.87,28.17) |
| 7 | Teferi et al [5] | 2022 | PMA | PMA survey | Cross-sectional | 678 | 9 | 33.33(29.78,36.88) |

Note: CI; confidence interval, SNNPR; Southern Nation Nationalities, and people region

**Prevalence of Homebirth Preference**

High heterogeneity was observed across the included studies (I2 = 98.2, $p < 0.001$) thus, a random-effects model was used to estimate the pooled prevalence of homebirth Preference. The prevalence of homebirth Preference was 39.62% (95% CI 27.98, 51.26). The highest 69.75(95% CI 64.98,74.51) prevalence of homebirth Preference was observed in Zone 3, Afar region [17] and the lowest 24.02(19.87,28.17) prevalence of homebirth Preference was reported in Arba Minch zuria surveillance site, SNNPR [21] (Fig. 2).

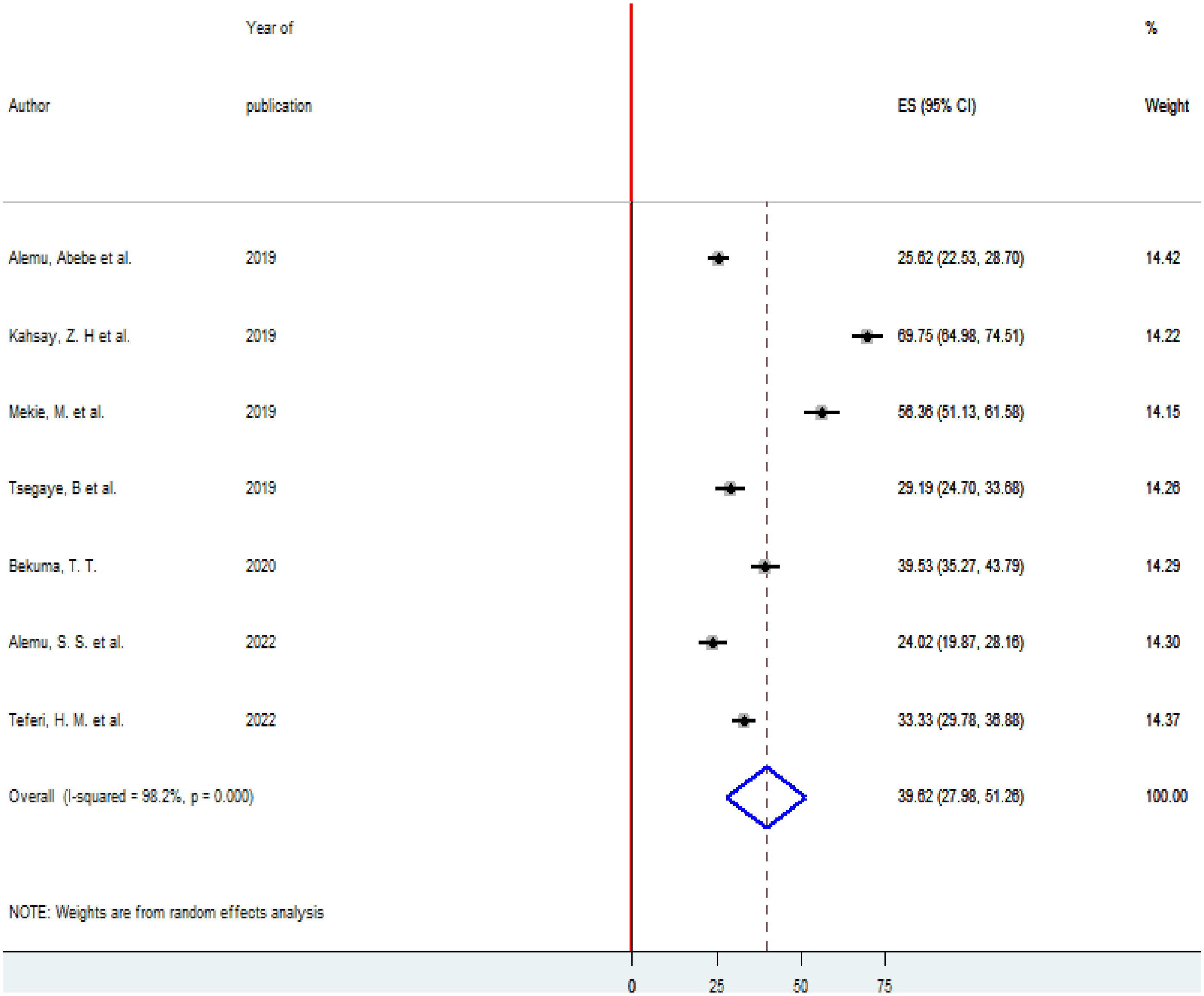

Fig. 2 Forest plot of the pooled estimate of the prevalence of Homebirth preference in Ethiopia, 2022

To check for underlying heterogeneity, meta-regression models were done by using sample size and year of publication, but there was statistically insignificant underlying heterogeneity (p = 0.309) and (p =0.421), respectively (Table 2).

**Table 2 Meta-regression analysis based on sample size and year of publication**

| Variables | Coefficients | p-value |
|---|---|---|
| Year of publication | -4.295715 | 0.421 |
| Sample size | -.0471067 | 0.309 |

**Subgroup analysis**

To see heterogeneity among the included studies, subgroup analysis was executed based on the area where the studies were conducted and the timing of studies (prospective and retrospective cross-sectional studies). According to where the studies conducted, the highest prevalence of homebirth Preference 47.49(95% CI 26.59,68.39) was observed in other (Oromia, Afar, and PMA survey data regions) [5, 17, 20] and the lowest prevalence of homebirth Preference 25.05(22.57,27.52) was reported in SNNPR (Fig. 3). According to the timing of the study, nearly the prevalence of homebirth Preference was equal among retrospective studies 40.4% (95% CI 23.28, 57.52) and prospective studies 39.04 (95% CI 20.47, 57.81) (Fig. 4).

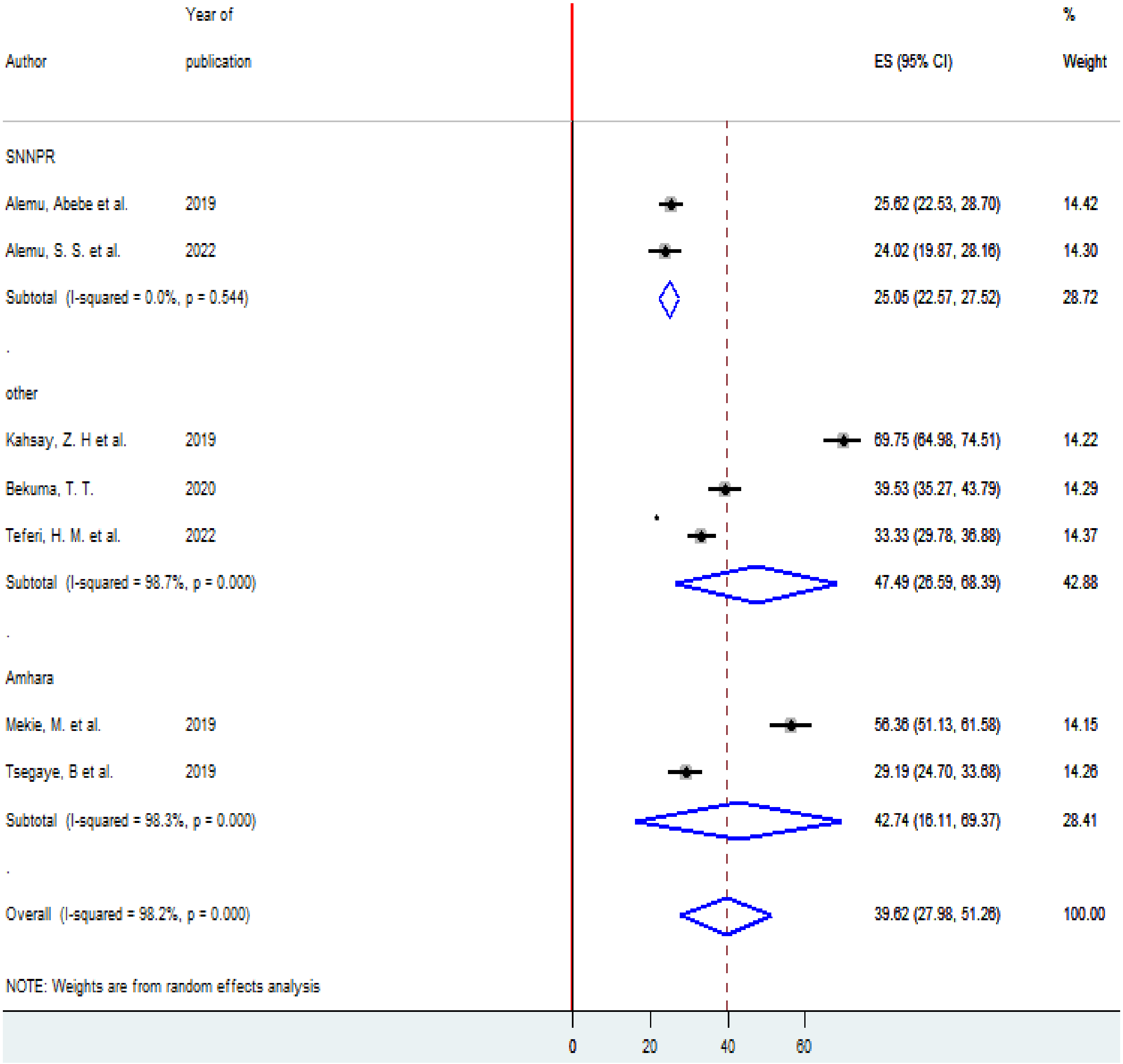


Fig. 3 Subgroup analysis of the prevalence of Homebirth preference among pregnant women in Ethiopia based on the region, 2022

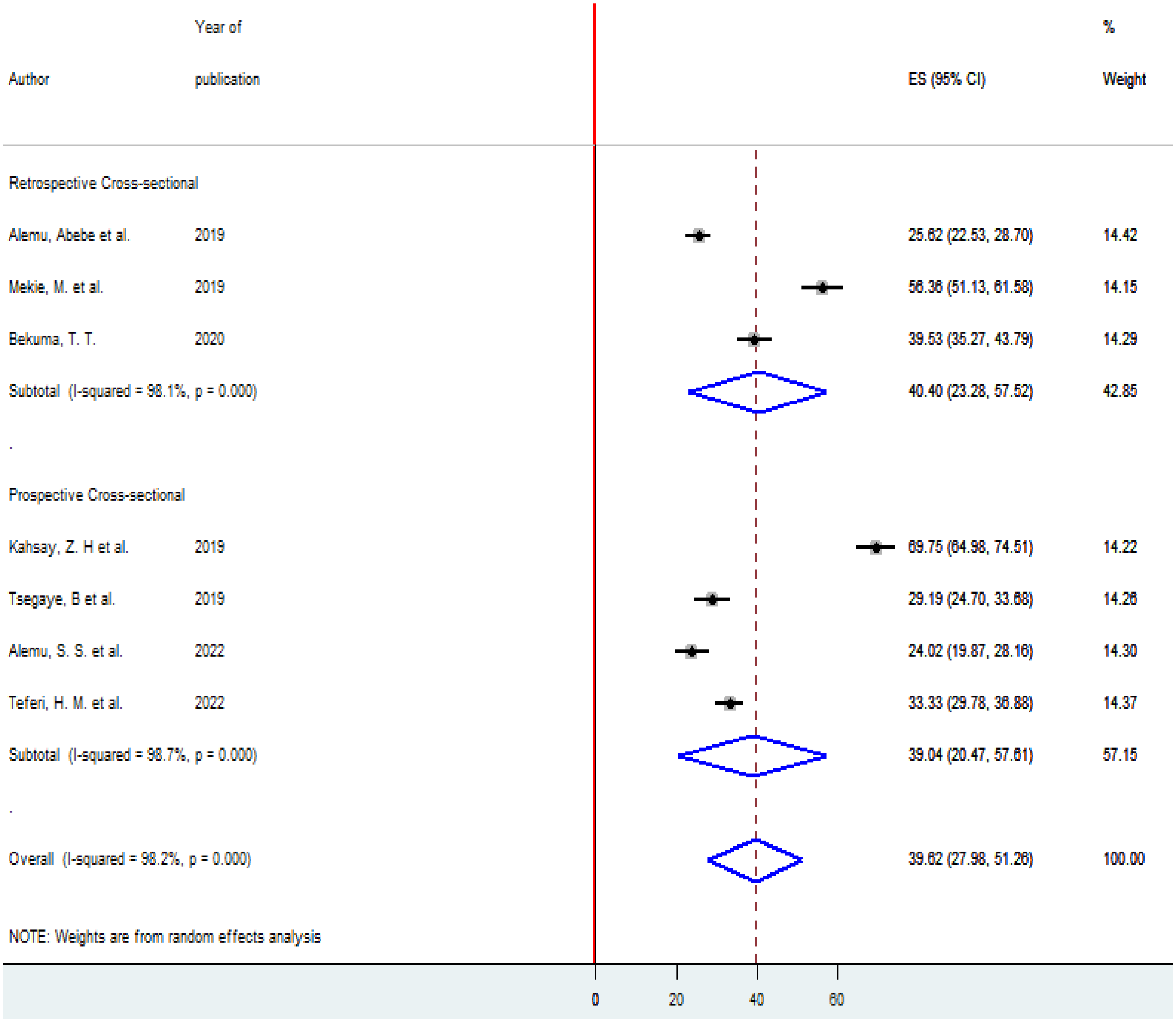

Fig. 4 Subgroup analysis of the prevalence of Homebirth preference among pregnant women in Ethiopia based on the timing of the study, 2022

## Publication bias

To see the presence of publication bias, the graphical funnel plot and Egger's test at a 5% significance level were executed. The visual examination of the funnel plot presented symmetrically which is an indicator for the absence of publication bias (Fig. 5). Egger's test also showed the absence of publication bias at a 5% significance level (p = 0.079).

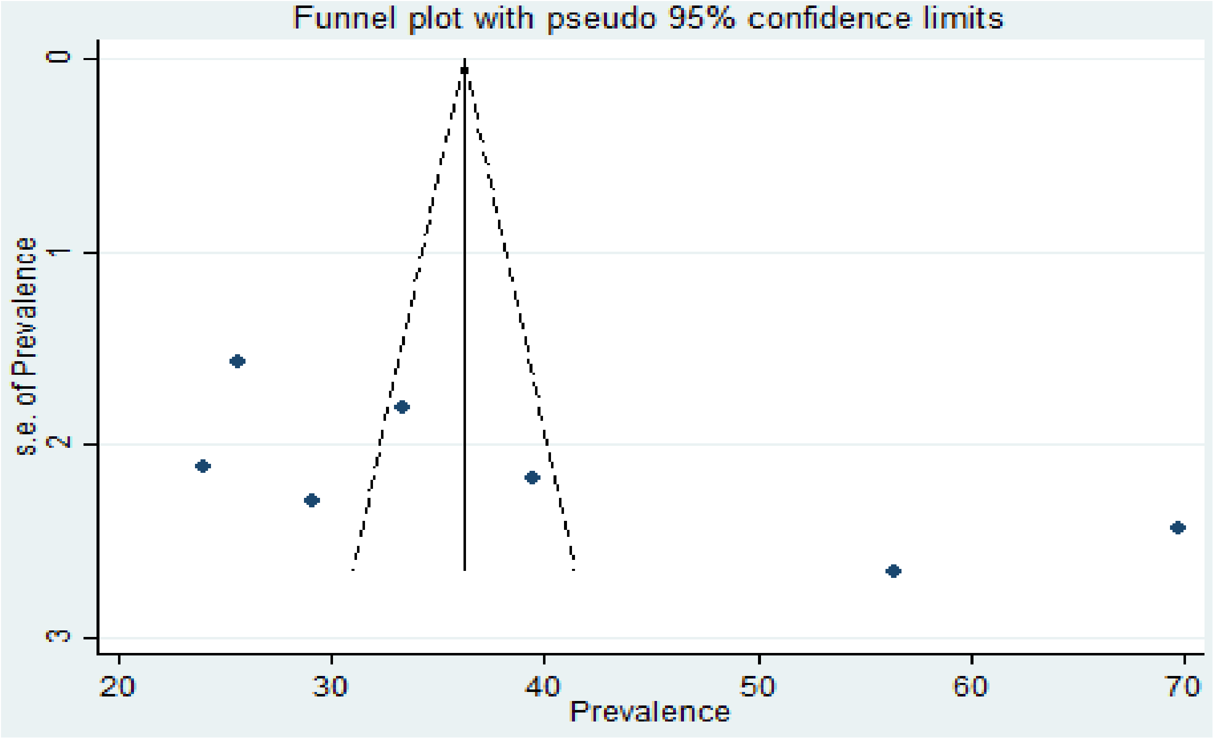


Fig. 5 Funnel plot with 95% confidence limit of the prevalence of Homebirth preference in Ethiopia, 2022

**Sensitivity analysis**

Sensitivity analysis was computed to see any outliers and it showed there was no single study influence on the overall included studies (Fig. 6).

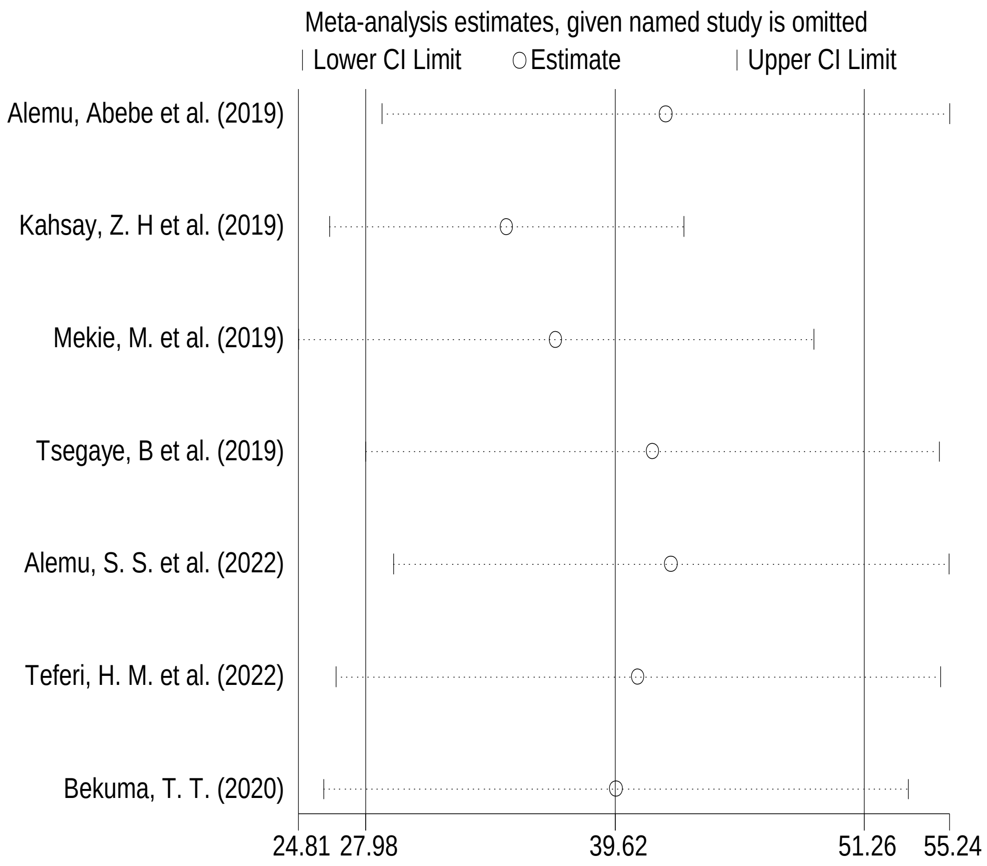


Fig. 6 Result of sensitivity analysis of homebirth preference in Ethiopia, 2020

## Factors associated with homebirth preference

### Association between Education and homebirth preference

To examine the association between maternal Education and homebirth preference, three studies were included in the analysis [10, 19, 20]. A random-effects model was used to estimate the pooled association between maternal education and homebirth preference ($I^2$ = 85.3%, P-value = 0.001). The pooled result of the analysis indicates that the association between maternal

education and homebirth preference was not statistically significant (OR = 0.72, CI 0.43, 1.19) (Fig. 7).

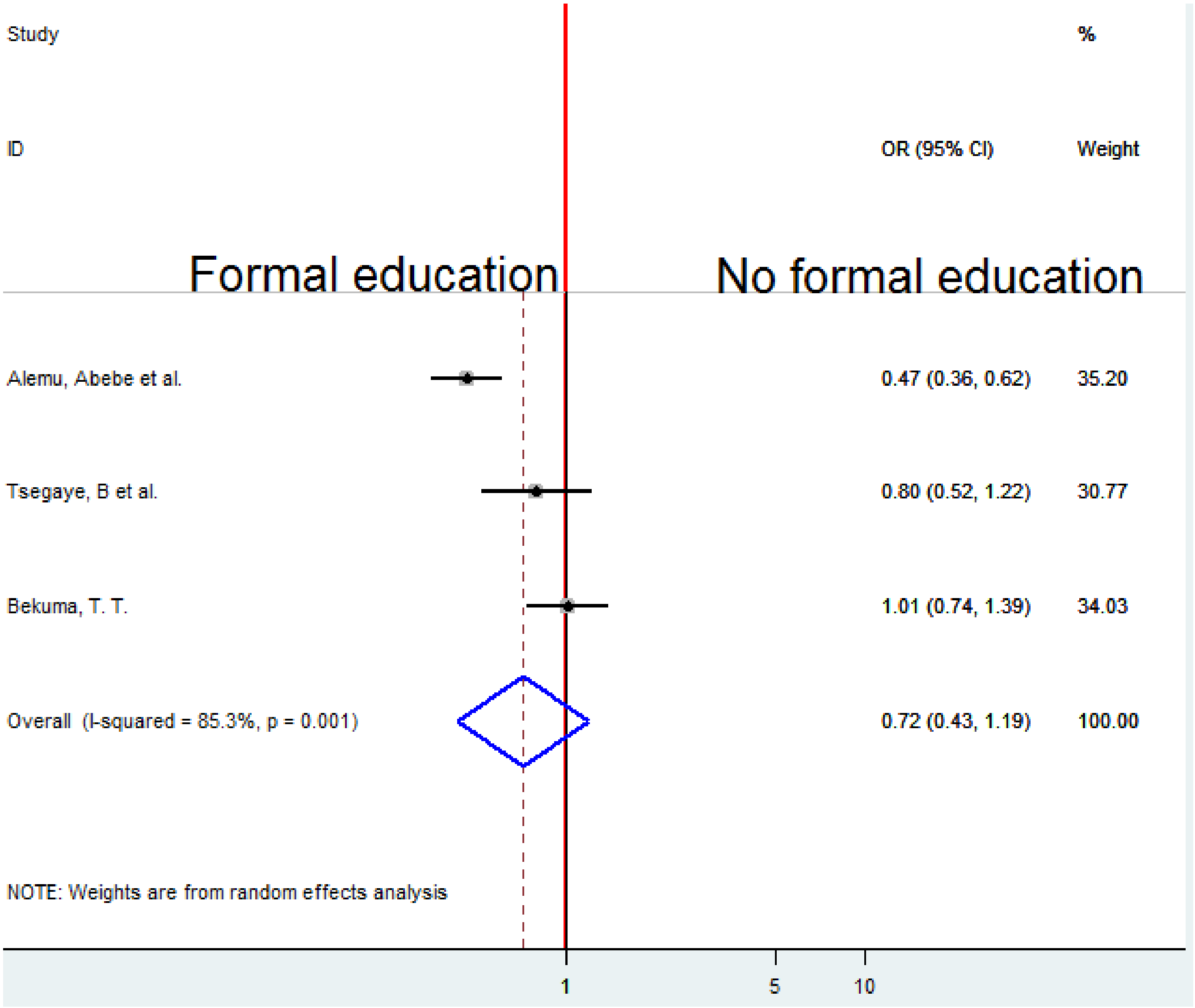


Fig. 7 Forest plot of the pooled estimate of the association between Education and homebirth preference in Ethiopia, 2022

**Association between monthly income and homebirth preference**

To examine the association between average monthly income and homebirth preference, two studies were included in the analysis [18, 19]. A random-effects model was used to estimate the pooled association between average monthly income and homebirth preference because of moderate heterogeneity (I2 = 69.0%). The pooled result of the analysis indicated that average monthly income < 1800 ETB was significantly associated with homebirth preference. Mothers who got <1800 ETB prefer homebirth 2.66 times more likely as compared to those who get 1800 ETB and above (OR = 2.66, 95% CI 1.44, 4.90) (Fig. 8).

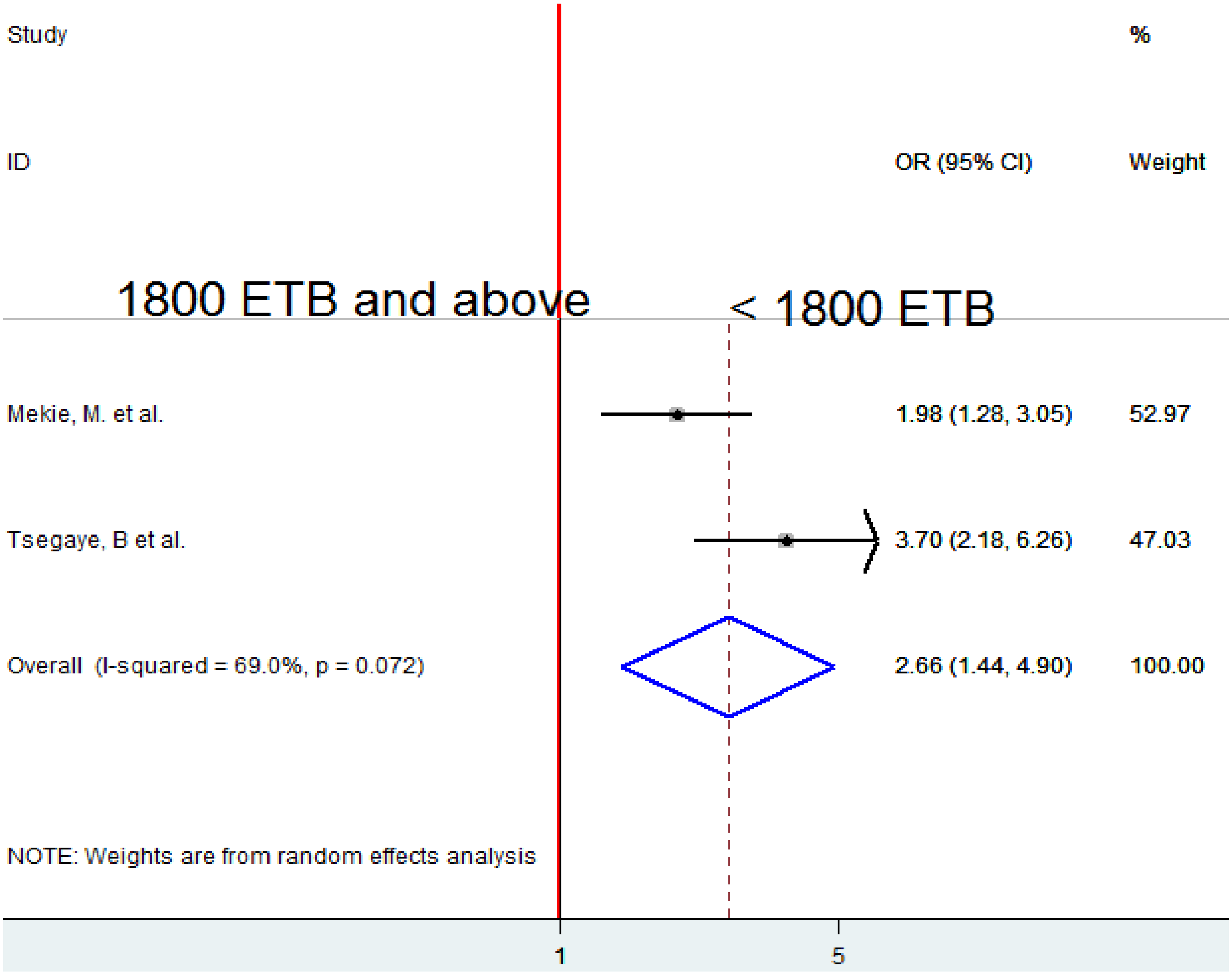

Fig. 8 Forest plot of the pooled estimate of the association between monthly income and homebirth preference in Ethiopia, 2022

## Association between ANC follow up and homebirth preference

To examine the association between ANC follow-up and homebirth preference, five studies were included in the analysis [5, 10, 18-20]. A random-effects model was used to estimate the pooled association between ANC follow-up and homebirth preference because of high heterogeneity (I2 = 88.1%, p-value < 0.001). The pooled result of the analysis indicates that not having ANC follow-up was significantly associated with homebirth preference. Women who did not have ANC follow-up preferred homebirth 2.57 times more likely as compared to those who had ANC follow-up during pregnancy (OR = 2.57, 95%CI 1.32, 5.01) (Fig. 9).

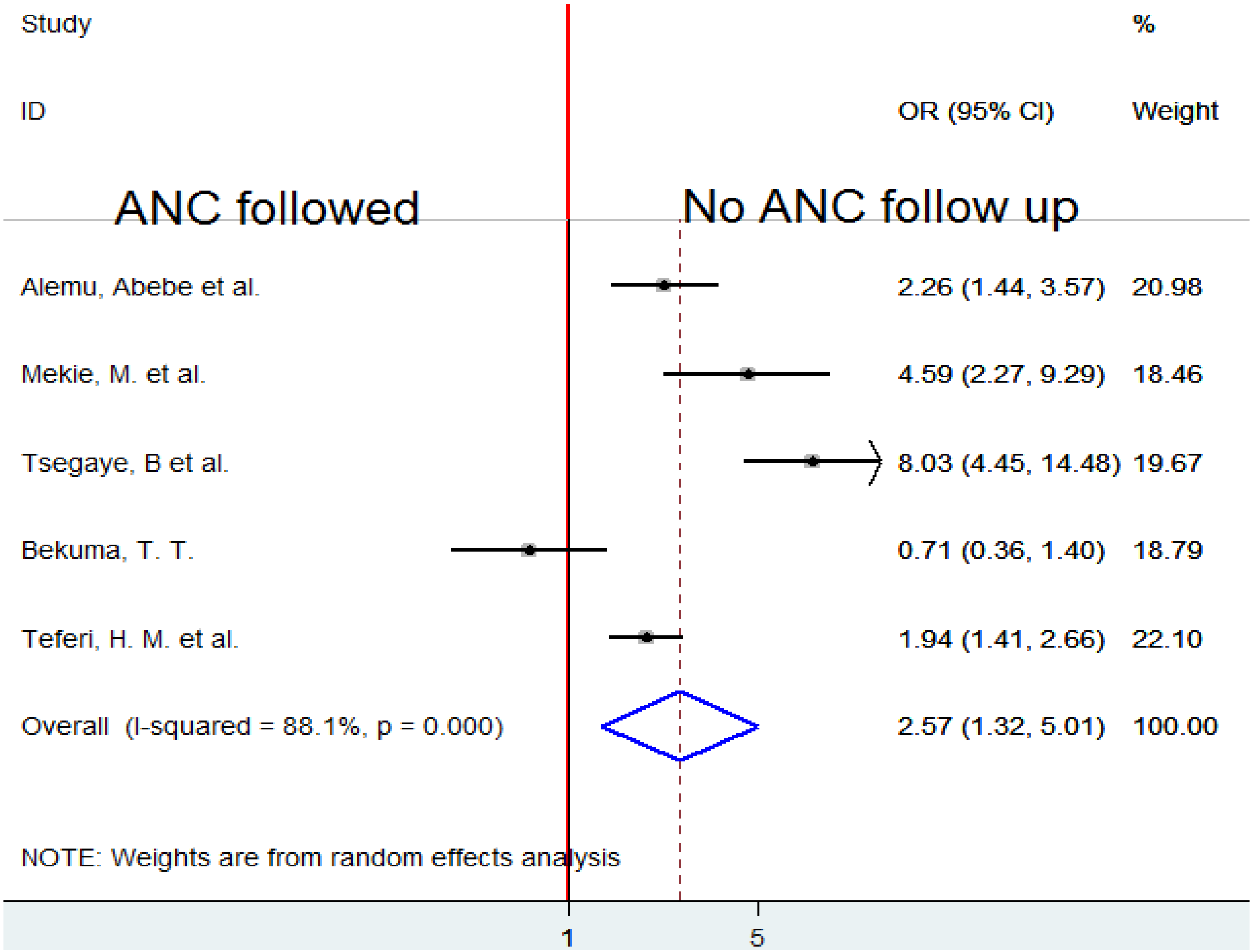


Fig. 9 Forest plot of the pooled estimate of the association between ANC follow up and homebirth preference in Ethiopia, 2022

**Association between Parity and homebirth preference**

To examine the association between parity and homebirth preference, two studies were included in the analysis [5, 10]. A fixed-effects model was used to estimate the pooled association between parity and homebirth preference because of no heterogeneity (I2 = 0 %, p-value < 0.576). The pooled result of the analysis indicates that parity was significantly associated with homebirth preference. Women of multipara prefer homebirth 1.77 times more likely as compared to those primiparas (OR = 1.77, 95%CI 1.39, 2.25) (Fig. 10).

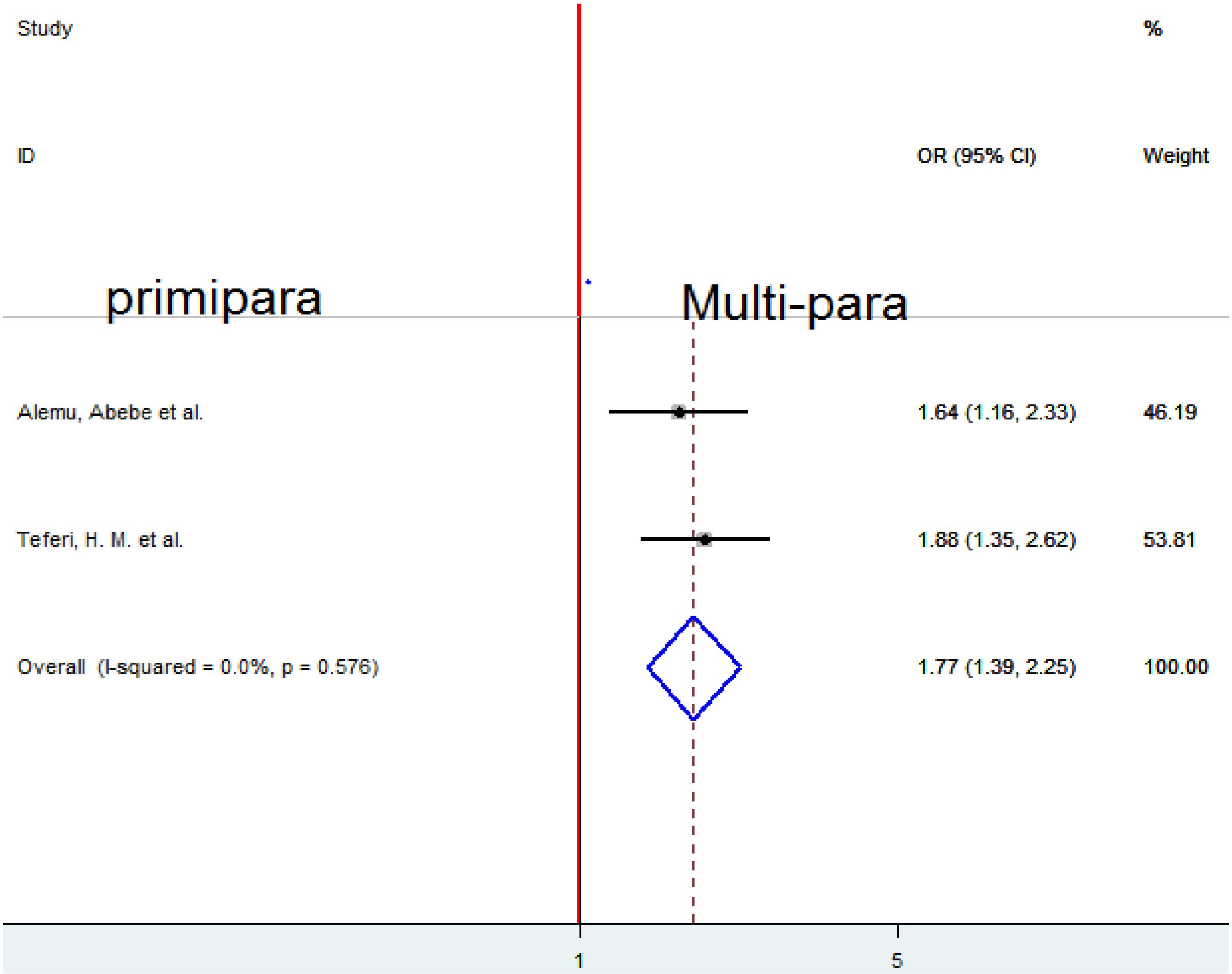


Fig. 10 Forest plot of the pooled estimate of the association between parity and homebirth preference in Ethiopia, 2022

**Association between knowledge of obstetric danger signs and homebirth preference**

To test the association between knowledge of obstetric danger sign follow-up and homebirth preference, two studies were included in the analysis [[10, 21]]. A random-effects model was used to estimate the pooled association between knowledge of obstetrics danger signs and homebirth preference because of high heterogeneity (I2 = 95.2 %, p-value < 0.001). The pooled result of the analysis indicates that poor knowledge was significantly associated with homebirth preference. Women who had poor knowledge preferred homebirth 5.75 times more likely as compared to those who knew obstetric danger signs (OR = 5.75, 95%CI 1.o2, 32.42) (fig.11)

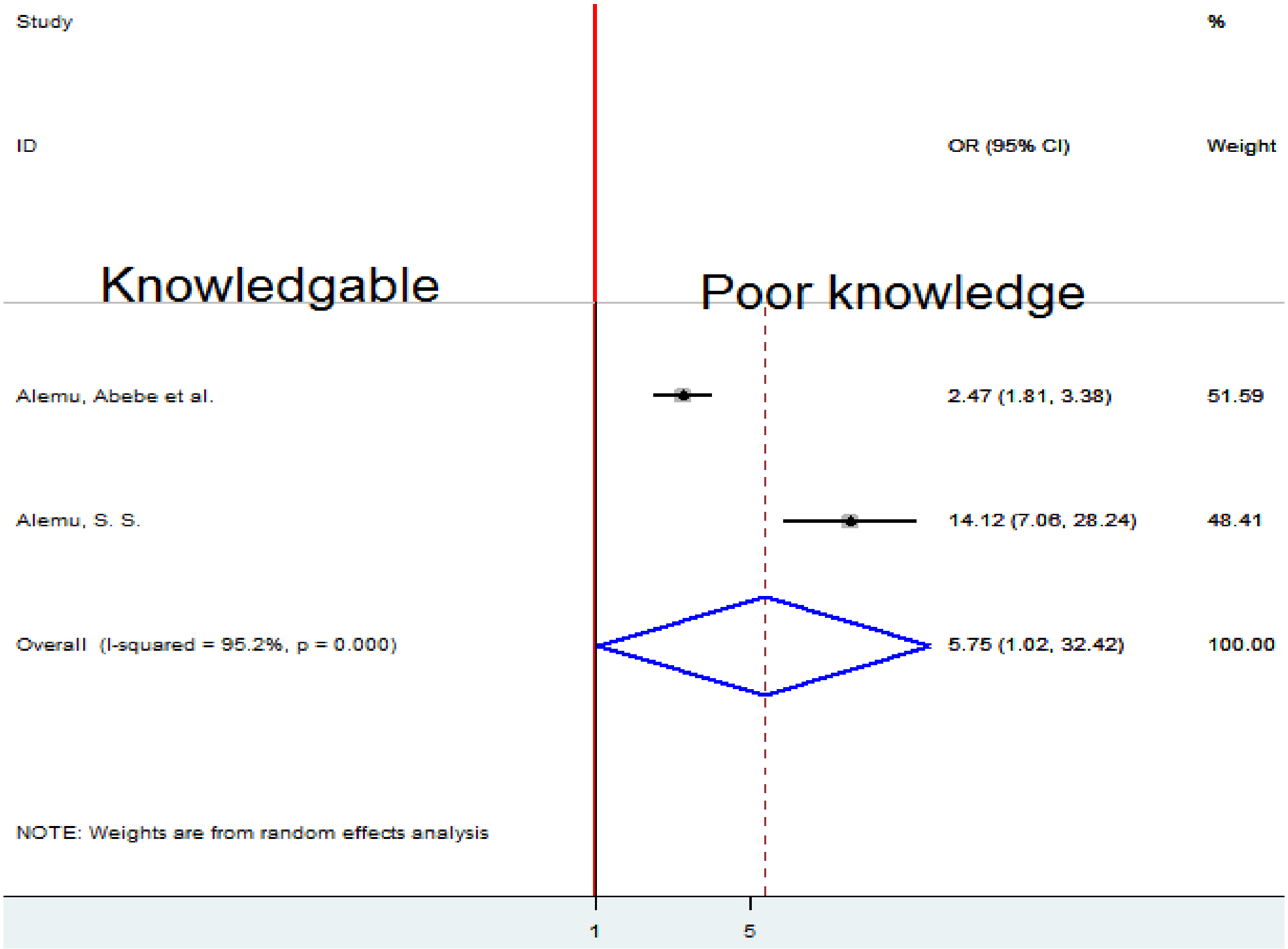

Fig.11 Forest plot of the pooled estimate of association knowledge about danger sign and homebirth preference in Ethiopia, 2022

**Association between transportation service accessibility and homebirth preference**

To examine the association between transportation service access and homebirth preference, three studies were included in the analysis [18, 20, 21]. A random-effects model was used to estimate the pooled association between ANC follow-up and homebirth preference because of high heterogeneity (I2 = 96.9 %, p-value < 0.001). The pooled result of the analysis indicated an insignificant association between transportation service access and homebirth preference. (Fig.12)**.**

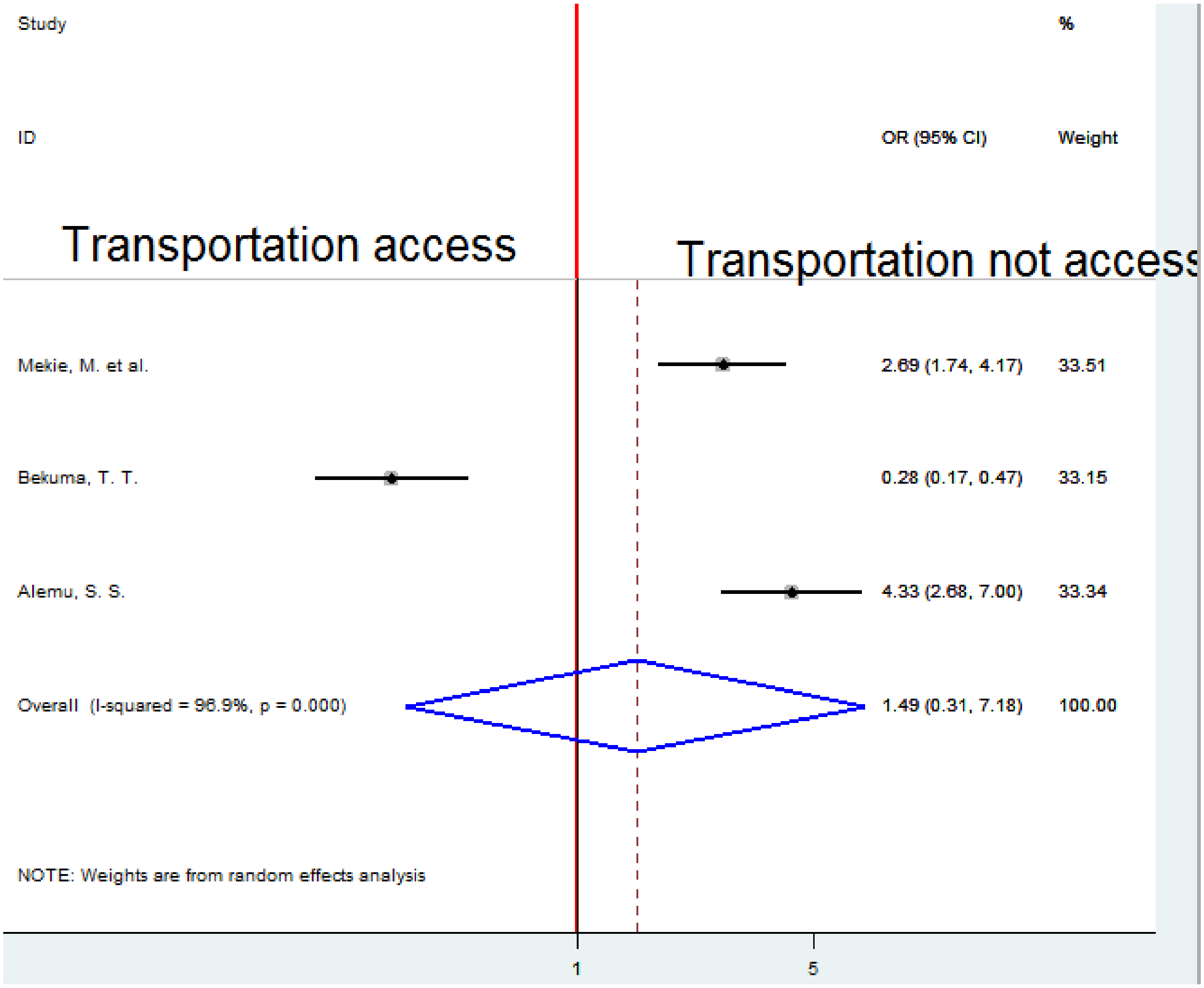


Fig. 11 Forest plot of the pooled estimate of the association between transportation access and homebirth preference in Ethiopia, 2022

**Association between delivery place discussion and homebirth preference**

To examine the association between delivery place discussion and homebirth preference, two studies were included in the analysis [5, 21]. A random-effects model was used to estimate the pooled association between delivery place discussion and homebirth preference because of high heterogeneity (I2 = 95.6%, p-value < 0.001). The pooled result of the analysis indicates that not having a delivery place discussion with a partner was significantly associated with homebirth preference.  Women who did not discuss with their partners a place of delivery prefer homebirth 5.89 (95%CI 1.1, 31.63) times more likely as compared to those who discussed with their partners (OR = 5.89 (95%CI 1.1, 31.63) (Fig. 12).

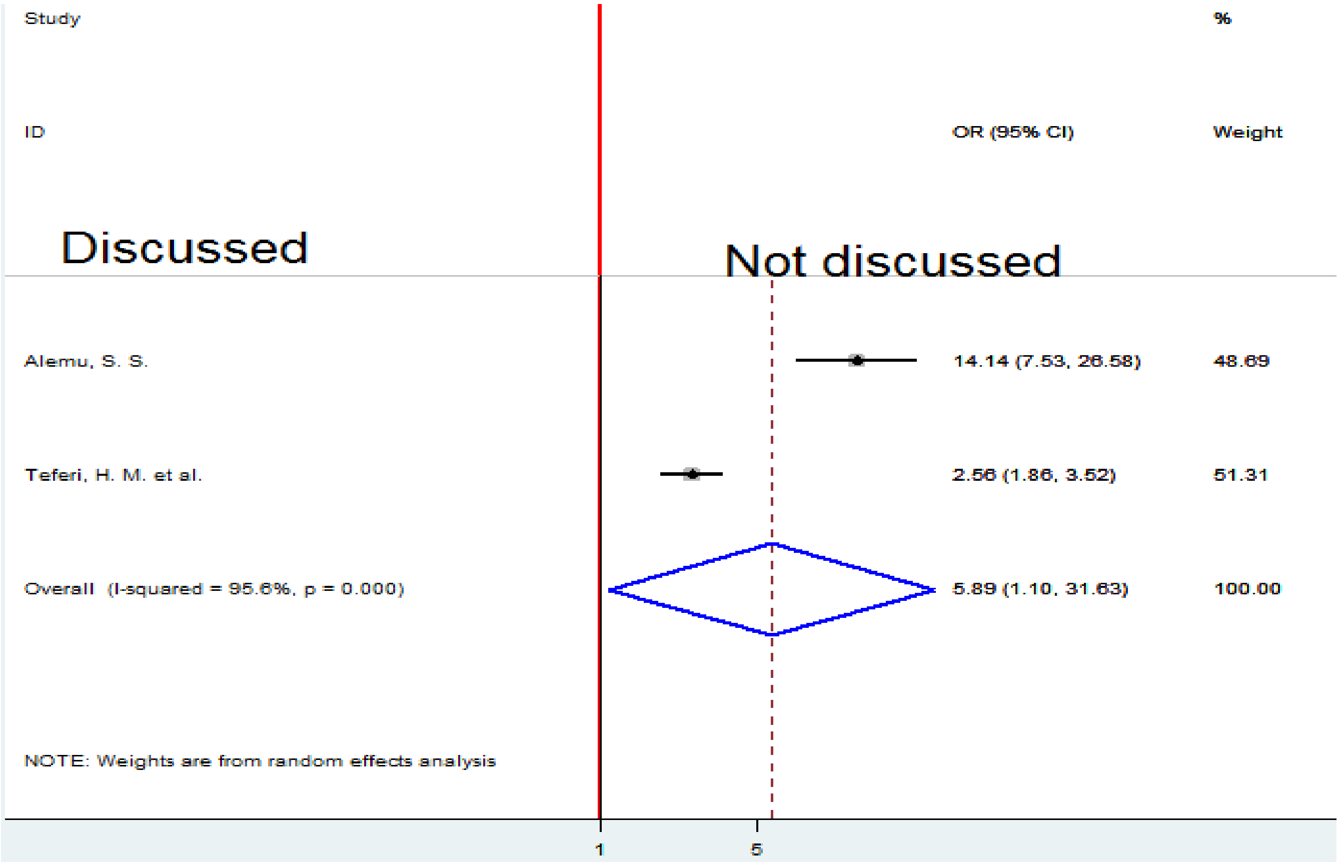


Fig. 12 Forest plot of the pooled estimate of the association between delivery place discussion and homebirth preference in Ethiopia, 2022

**Association between the place of last delivery and homebirth preference**

To examine the association between the place of last delivery and homebirth preference, two studies were included in the analysis [10, 18]. A random-effects model was used to estimate the pooled association between the place of last delivery and homebirth preference because of high heterogeneity (I2 = 97.2 %, p-value < 0.001). The pooled result of the analysis indicated an insignificant association between transportation service access and homebirth preference (Fig.13).

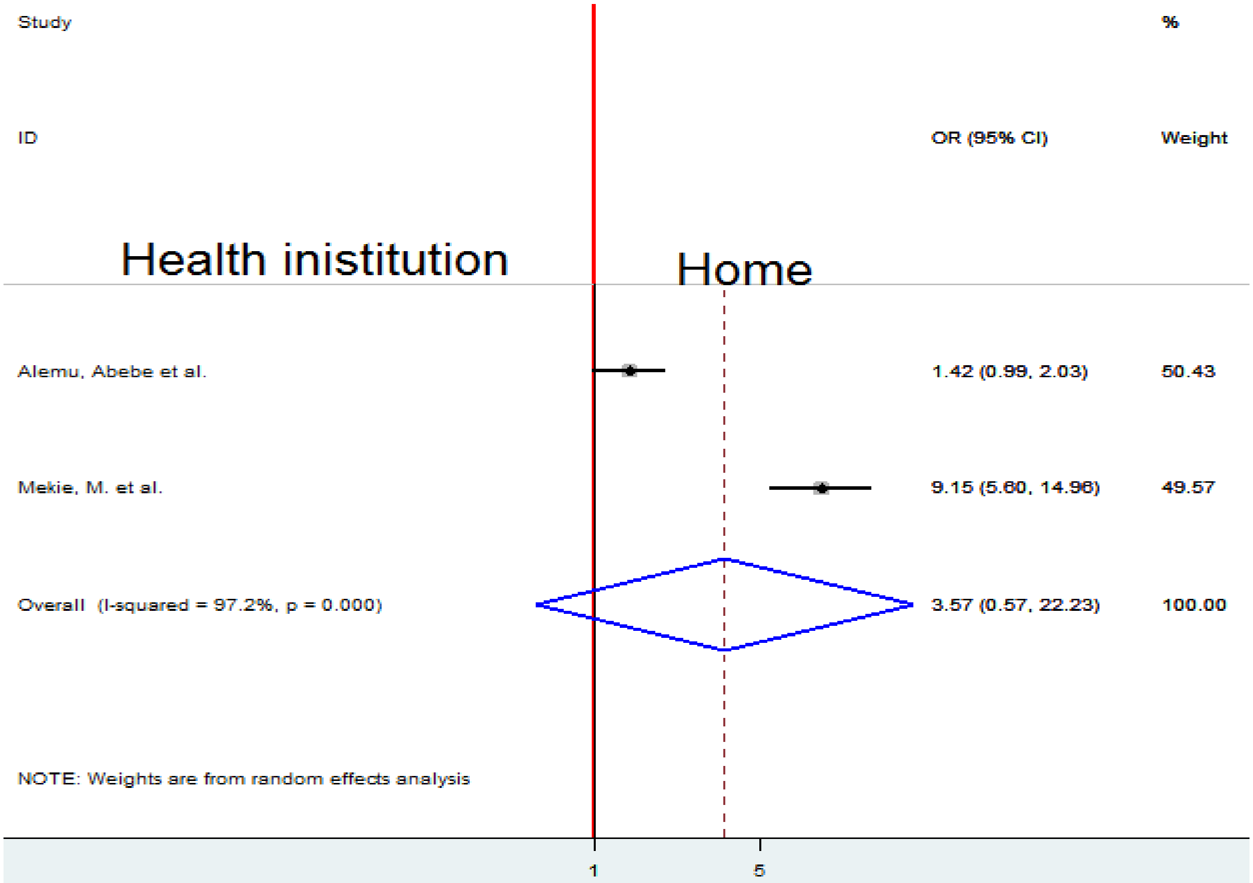

Fig. 13 Forest plot of the pooled estimate of the association between place of last delivery and homebirth preference in Ethiopia, 2022

## DISCUSSION

This systematic review and meta-analysis assessed homebirth preference and associated factors in Ethiopia, 2022.

The prevalence of homebirth preference among pregnant women in Ethiopia was 39.62% (95% CI 27.98, 51.26). Even though, WHO and EFMOH inspire every woman to give birth at a health institution this systematic review and meta-analysis revealed that the prevalence of homebirth preference is high. This finding is consistent with a study done in Nigeria 39.3% [22]. But the finding of this systematic review and meta-analysis is higher than the finding of the study conducted in Tanzania (25.5%) [23]. A possible explanation for this inconsistency finding might be due to the local versus national study populations involved in these studies. The discrepancy could be due to the difference in the study subjects, the previous study was undertaken among all reproductive women whereas most of the participants of this systematic review and meta-analysis were pregnant mothers. Pregnant women's favorite for homebirth declines as gestational age upturns because most pregnant women start ANC follow-up after the second trimester therefore they get advice and counseling about the benefit of institutional delivery.

This systematic review and meta-analysis revealed that low average monthly income (< 1800 ETB) was significantly associated with homebirth preference. Women with low average monthly income were about 3 fold times more likely to prefer homebirth than their counterparts. This finding is consistent with the study done in Tanzania [24]. Health care in Ethiopia for pregnant women is free. The reason might be healthcare facilities seldom run out of stock, demanding pregnant mothers to charge some expenses for supplies or services. Besides, women from distance pay a transportation fee to go to a health facility for maternity care. Therefore, pregnant women with better incomes are better to pay for transport and other health care payments.

Knowledge about obstetrical danger signs is significantly associated with home birth preference. Homebirth preference among women who had poor knowledge about danger signs was about 6 fold times higher compared to knowledgeable women. This is supported by the findings of studies undertaken in Ghana [25]. The possible explanation might be maternal education and counseling which is given during ANC follow up can change and boost the knowledge of pregnant mothers. As the result, this systematic review and meta-analysis indicated fail to attend ANC was significantly associated with homebirth preference. This result is revealing that the one who has ANC follow-up can have advice and counseling regarding obstetric danger signs. This can enhance women's intention to give birth at the health institution. As the knowledge of women about obstetric danger signs is boosted, understanding the benefits of health institution delivery becomes more efficient than homebirth.

In this systematic review and meta-analysis, multipara mothers were about 2 fold times more likely to prefer homebirth compared to nulliparous women in Ethiopia. This finding is in line with a study conducted in Ghana [26]. A possible explanation may be that compared to the nulliparous, multiparas are usually self-possessed regarding homebirth because of their previous childbirth understanding.

Mothers not attended ANC visits in the current pregnancy were about 3 fold times more likely to prefer home delivery than those who attended ANC in Ethiopia. This finding is consistent with studies conducted in Nigeria [22]. It might be women who had ANC visits by health professionals had better opportunities to be counseled regarding birth preparedness, complication readiness, and place of delivery which may enhance delivery at a health institution than preferring to give birth at their own home.

According to this systematic review and meta-analysis, women who did not discuss the place of delivery with their partners were about 6 fold times more likely to prefer homebirth compared to those who discussed it with their partner. Previous studies done in Tanzania and Mozambique have shown consistent findings [27]. The possible explanations for this finding might be, if the pregnant woman is financially dependent on her partner and the partner does not know the advantage of Institutional delivery he can decide the place of delivery by himself as he is the source of finance. If couples are involved in a discussion on the place of delivery, the probability to know the benefit and side effects of homebirth might be discussed. When partners shared that the burden might come to them due to homebirth they may decide to give birth at a health institution. Thus why, not discussing with a partner may result in homebirth.

In this systematic review and meta-analysis, the pooled find of the educational status of mothers, transportation access, and place of last delivery were not statistically significant with homebirth preference (Fig 7, 11, 13).

**Strength and limitation**

This systematic review and meta-analysis has strengths; various databases were used to search literature, both published and unpublished studies were searched and relevant studies were included after intensive quality assessment. The limitations of the review; studies included in the review were only published in the English language which may limit the number of studies; those were reported in other languages. Second, only cross-sectional studies were included, which may decrease causal conclusions between homebirth preference and associated factors. Because of less than 10 studies were included in the final analysis, it is difficult to get the exact estimation of publication bias from the funnel plot.

## Conclusion

This systematic review and meta-analysis examined the high prevalence of homebirth preference. This high prevalence of homebirth preference was associated with low average monthly income (<1800 ETB), lack of ANC follow-up, multipara, poor knowledge about obstetric danger signs, and not discussing with their partner a place of delivery. Furthermore, a discrepancy was also found in different regions of the country. So, Community based awareness creation is mandatory to improve ANC follow-up. The health policy of the country should include a mechanism that encourages, couples to discuss together the exact choice of the right place of delivery to minimize perinatal and postpartum complications. Community health workers in the country should routinely deliver the basic knowledge of obstetric danger signs for pregnant mothers at a community level.

## Abbreviations

ANC: Antenatal care; CI: Confidence interval; OR: Odd ratio; SNNPR: Southern Nation nationality and people region; WHO: World Health organization; EFMOH: Ethiopian Federal Ministry of Health

## Acknowledgments

We would like to thank all authors of the studies included in this systematic review and meta-analysis.

## Author contributions

JW, EM, ML, WB, MD, AT, DD, GF, RD, SS and KM were involved in the design, statistical analysis, manuscript writing, and participated in the selection of articles and data extraction. All

authors were involved in developing the initial drafts of the manuscript, revising subsequent drafts, and preparing the final draft of the manuscript. All authors read and approved the final manuscript.

**Funding**

No funding was obtained for this study.

**Availability of data and materials**

The datasets analyzed during the current review and meta-analysis are available from the corresponding author upon reasonable request.

**Declarations**

**Ethics approval and consent to participate**

Not applicable.

**Consent for publication**

Not applicable.

**Competing interests:** The authors declare that they have no competing interests.

**Author Details**

1. Department of Public Health, Institute of Health Sciences, Wollega University, P.O.BOX: 395, Nekemte, Ethiopia.

2. Department of Nursing, College of Health Sciences, Mettu University, Mettu, Ethiopia

3. Department of Nursing, Institute of Health Sciences, Wollega University, P.O.BOX: 395, Nekemte, Ethiopia

4. Department Midwifery, College of Health Sciences, Madda Walabu University, Shashamane, Ethiopia

5, Department of Public Health, College of Health Sciences, Mettu University, Mettu, Ethiopia

## Reference


1. Shiferaw, S., et al., *Why do women prefer home births in Ethiopia?* BMC Pregnancy and Childbirth, 2013. **13**(1): p. 5.
2. Cofie, L.E., et al., *Birth location preferences of mothers and fathers in rural Ghana: Implications for pregnancy, labor and birth outcomes.* BMC Pregnancy and Childbirth, 2015. **15**(1): p. 165.
3. Fantaye, A.W., N. Gunawardena, and S. Yaya, *Preferences for formal and traditional sources of childbirth and postnatal care among women in rural Africa: a systematic review.* PloS one, 2019. **14**(9): p. e0222110.
4. Aychiluhm, S.B., et al., *Geographical clustering and geographically weighted regression analysis of home delivery and its determinants in developing regions of Ethiopia: a spatial analysis.* Emerging Themes in Epidemiology, 2022. **19**(1): p. 8.
5. Teferi, H.M., M. San Sebastian, and M. Baroudi, *Factors associated with home delivery preference among pregnant women in Ethiopia: a cross-sectional study.* Glob Health Action, 2022. **15**(1): p. 2080934.
6. Ethiopian Public Health Institute - EPHI, Federal Ministry of Health - FMoH, and ICF, *Ethiopia Mini Demographic and Health Survey 2019*. 2021, EPHI/FMoH/ICF: Addis Ababa, Ethiopia.
7. Nigusie, A., et al., *Community perception of barriers and facilitators to institutional delivery care-seeking behavior in Northwest Ethiopia: a qualitative study.* Reproductive Health, 2022. **19**(1): p. 193.
8. Mutharayappa, K. and P. Prabhuswamy, *Factors and Consequences of Home Deliveries: A Study in Rural Karnataka.* Journal of Health Management, 2003. **5**(1): p. 17-35.
9. Kolin, D.A., et al., *Risk factors for blood transfusion in traumatic and postpartum hemorrhage patients: Analysis of the CRASH-2 and WOMAN trials.* PLOS ONE, 2020. **15**(6): p. e0233274.
10. Alemu, A., G. Amenu, and M. Feyiso, *Women's preference of home delivery in Wonago District, Gedeo Zone, Southern Ethiopia 2018.* J Gynecol Obstet, 2019. **7**(3): p. 85.
11. Moher, D., et al., *Guidelines and Guidance-Preferred Reporting Items for Systematic Reviews and Meta-Analyses: The PRISMA Statement.* PLoS Medicine, 2009. **6**(7): p. 698.
12. Moher, D., et al., *Preferred Reporting Items for Systematic Reviews and Meta-Analyses: The PRISMA Statement.* PLOS Medicine, 2009. **6**(7): p. e1000097.
13. Institute, J.B., *Meta-analysis of statistics: Assessment and review instrument (JBI MASTARI).* Adelaide: Joanna Briggs Institute, 2006. **20032007**.
14. Luchini, C., et al., *Assessing the quality of studies in meta-analyses: Advantages and limitations of the Newcastle Ottawa Scale.* World J Meta-Anal, 2017. **5**(4): p. 80-84.
15. Higgins, J.P. and S.G. Thompson, *Quantifying heterogeneity in a meta-analysis.* Stat Med, 2002. **21**(11): p. 1539-58.
16. Begg, C.B. and M. Mazumdar, *Operating characteristics of a rank correlation test for publication bias.* Biometrics, 1994. **50**(4): p. 1088-101.
17. Kahsay, Z.H., et al., *Pregnant Women's Intentions to Deliver at a Health Facility in the Pastoralist Communities of Afar, Ethiopia: An Application of the Health Belief Model.* Int J Environ Res Public Health, 2019. **16**(5).
18. Mekie, M. and W. Taklual, *Delivery place preference and its associated factors among women who deliver in the last 12 months in Simada district of Amhara Region, Northwest Ethiopia: a community based cross sectional study.* BMC Res Notes, 2019. **12**(1): p. 114.
19. Tsegaye, B., et al., *Level and factors associated with preference of institutional delivery among pregnant woman in Debre-tabor town, North West Ethiopia, 2017: a community based cross sectional study.* BMC Res Notes, 2019. **12**(1): p. 44.

20. Bekuma, T.T., et al., *Factors Affecting Choice of Childbirth Place among Childbearing Age Women in Western Ethiopia: A Community-Based Cross-Sectional Study.* Int J Reprod Med, 2020. **2020**: p. 4371513.
21. Alemu, S.S., et al., *Preference of homebirth and associated factors among pregnant women in Arba Minch health and demographic surveillance site, Southern Ethiopia.* PLoS One, 2022. **17**(10): p. e0276682.
22. Envuladu, E., et al., *Factors determining the choice of a place of delivery among pregnant women in Russia village of Jos North, Nigeria: achieving the MDGs 4 and 5.* International Journal of Medicine and Biomedical Research, 2013. **2**(1): p. 23-27.
23. Exavery A, K., Njozi M, Tani K, Doctor HV, Hingora A, Phillips *Access to institutional delivery care and reasons for home delivery in three districts of Tanzania.* International journal for equity in health. , 2014. **13**(1): p. 48.
24. Ngowi, A.F., et al., *Women's determinant factors for preferred place of delivery in Dodoma region Tanzania: a cross sectional study.* Reproductive Health, 2017. **14**(1): p. 112.
25. Nakua, E.K., et al., *Home birth without skilled attendants despitemillennium villages project intervention in Ghana: insight from a survey of women's perceptions of skilled obstetric care.* BMC Pregnancy and Childbirth 2015. **15**: p. 243.
26. Boah, M., et al., *"I couldn't buy the items so I didn't go to deliver at the health facility" Home delivery among rural women in northern Ghana: A mixed-method analysis.* PLOS ONE, 2020. **15**(3): p. e0230341.
27. Mpembeni RNM, K.J., Leshabari MT, et al., *Usepattern of maternal health services and determinants of skilled care during delivery in Southern Tanzania: implications for achievement of MDG-5 targets.* BMC Pregnancy Childbirth, 2007. **7**(1-7).

Click here to access/download
**Supporting Information**
PRISMA_checklist.docx
