# Supplementary material for: Prevalence of homebirth preference and associated factors among pregnant women in Ethiopia: Systematic review and meta-analysis
Source: PLoS One. 2023 Nov 15;18(11):e0291394. doi: 10.1371/journal.pone.0291394 (PMC10651027; doi:10.1371/journal.pone.0291394)
Supplement: Attachment — Submitted filename: Plos.docx [file pone.0291394.s003.docx]

**Manuscript #:** PONE-D-23-02877
**Title:** PREVALENCE OF HOMEBIRTH PREFERENCE AND ASSOCIATED FACTORSAMONG PREGNANT WOMEN IN ETHIOPIA: SYSTEMATIC REVIEW AND META-ANALYSIS
**Article type:** Research Article

Comments to authors

**Abstract:**

Background:

What is the problem? Is it homebirth itself or not being attended by skilled birth attendant?

Methods:

All parts of method section should be summarized. An important time here is years of publications that included not the time that you conducted searching. Use of funnel plot is subjective.
Conclusion: what is the findings implication?

Background

Authors should indicated contextual differences comparing different geographical, economic and social backgrounds, i.e., global to local.

Why should we care about homebirth? What proportion of morbidity mortality are attributable to home birth?

“*Even though Ethiopia has devised Critical strategies including promoting institutional delivery services for lowering maternal morbidity and mortality, just 1 in 4 women gave birth in a medical facility in the country [4]*.” But the current data in Ethiopia different from this! Update your source of evidence.

“*According to the 2019 Mini Ethiopian Demographic and Health Survey report, of all live births in the five years before the survey, only 50% were delivered by a skilled provider [6].*” Don’t you think this contradict with above report?

Show the controversies of findings of the studies behind the variation you evidenced/confirmed. In other word, where is/are the variation(s)?

“*Therefore, this systematic review and meta-analysis aimed to provide more current and representative national data on homebirth Preference and associated factors in Ethiopia*.”

How this provide current data? You pooled already available evidences.

Method

Include PROSPERO registration number.

“The research articles search strategy, selection of studies, data extraction, and result reporting were done in accordance with Preferred Reporting Items for Systematic Reviews and Meta-Analyses (PRISMA) guidelines [11]. A PICO principle was adapted for searching terms. (PRISMA) guidelines were used to state this systematic review and meta-analysis [12].” What the difference between these two references? Which PRISMA guideline was used in your case?

What PICO includes in your case?

Explain databases and non-databases’ search engine separately.

What is the difference between Web of Science and WoS?

How do authors make sure search terms are not missed?

Include sample searching strategy for at least one database. The searching strategies for other databases should be annexed as additional files with its appropriate citation here.

Eligibility:

Clearly indicate the eligibility criteria using bullet or numbering.

*“…..included all studies that were conducted on homebirth preference and associated factors among pregnant women in Ethiopia*.” what if your outcome and factors are missing?

“*The participants were pregnant women/mothers*” what do you mean? Did you conduct primary study?

“*We included all types of articles that were published in the form of journal articles, master's theses, and dissertations in the English language. Published studies were included in the study, but unpublished studies were not found.*” This is eligibility criteria, it is not where you report availability of the unpublished articles. Further, make sure the sentences are clear to audience.

“*Full research articles, which were not accessed after at least two email contacts of the primary author, were not included because of the failure to assess the quality of articles in the absence of full text.*” I failed to understand the meaning and importance of this sentence.

What is timeframe for included studies? Why studies before 2016 excluded? However, Ethiopia was striving to improve institutional delivery during MDG. The institutional delivery increased from 6% to

“*All studies conducted in Ethiopia were included*.” This is default.

Outcome

Is the content of this paragraph is only about outcome variables? Rename subtopic.

“*The first outcome of interest was the prevalence, which was estimated as the total number of women preferring homebirth, cases divided by the total number of sample sizes multiplied by 100.*” Do you mean? How ‘prevalence of home birth preference’ calculated? Did you pool calculated proportion or pool all events and samples to calculate pooled proportion?

Clearly state how you measured all variables (outcome and explanatory).

Data collection and quality assessment

Is ‘data collection’ appropriate here?

What are finding of the Joanna Briggs Institute? How many articles were excluded due to quality? How?

“*Three data extractors (JW, EM and ML) extracted data by using a standardized data extraction checklist on Microsoft Excel*.” Authors should mention the content of checklist that used for data extraction.

The authors should separately write the data extraction and quality control clearly. Who did the data extraction? And how about the quality of included articles?

“*Inconsistencies between three independent reviewers were fixed by including other reviewers (MD, AT, WB and KM) after discussion for possible agreement.*” How do think this can happen?

Data analysis and synthesis

“*Using a format prepared in a Microsoft Excel spreadsheet, necessary information from each original study was extracted*.” You already told us above! Why you repeat same thing here and there. This comments works in many place in your document.

Have conducted sensitivity analysis? How?

“*Meta-regression was computed to see the presence of statistically significant heterogeneity.*” What is the purpose of meta-regression? When do you conduct it?

Result

Use standard PRISMA flow chart. This is not attractive to audience.

Separately mention articles that obtained from databases and additional sources.

How these three articles can be included if your search is limited with timeframe from the very beginning?

Table 1 How about study setting?

Prevalence of Homebirth Preference:

Why do you think there is high heterogeneity?

“*The highest 69.75(95% CI 64.98,74.51) prevalence of homebirth Preference was observed in Zone 3, Afar region [17] and the lowest 24.02(19.87,28.17) prevalence of homebirth Preference was reported in Arba Minch zuria surveillance site, SNNPR* [21]” what do you mean? Are these the pooled prevalence?

Figure 2 has small font size and difficult to read.

What aspects of variations should you consider to bring down this high heterogeneity in addition to sample size and publication year?

Publication bias

“*The visual examination of the funnel plot presented symmetrically which is an indicator for the absence of publication bias (Fig. 5*)” this funnel plot is not symmetry.

Sensitivity analysis

Have you observed the output after excluding Kaysay et al., 2019 article? Authors need to be honest when reporting findings. Tell the audience what you found with and without Kaysay *et al*., 2019 article.

Factors associated with homebirth preference

In all figures exposure and non-exposure were not appropriately placed. Indicate exposure and non-exposure status in all figures.

Generally the figures need edition to make them attractive to audience. The result sections are full of unnecessary statements and lack focus.

Discussion

The explanations that given to similarities and differences need appropriate and proper citation. The author repeated result in discussion. The discussion lack focus.

Conclusion

Same comment as in abstract.
