# Supplementary material for: Prevalence of homebirth preference and associated factors among pregnant women in Ethiopia: Systematic review and meta-analysis
Source: PLoS One. 2023 Nov 15;18(11):e0291394. doi: 10.1371/journal.pone.0291394 (PMC10651027; doi:10.1371/journal.pone.0291394)
Supplement: Attachment — Submitted filename: Response to reviewers and editor.docx [file pone.0291394.s005.docx]

**Responses to Editor and Reviewers**

First of all, we would like to thank the Editor and Reviewers of this manuscript for giving us such constructive comments and questions to enrich this manuscript.

| S/N | Questions/comments | Authors response |
| --- | --- | --- |
|  | **General comments** |  |
| 1 | The language of the manuscript needs to be improved for publication. | Language has been revised |
| 2 | The problem studied is not well explained in a focused manner? | Modified with clear explanation of the problem |
| 3 | I am not convinced why only studies published after 2016 are included? I recommend you to include those 3 studies published before 2016. | The reason is that the research articles published before 2016 were not representative of the current strategy/policy of the country. This means the reality of the country, the current strategy which is from 2016 is the focused strategy to reduce home birth preference to reduce maternal mortality occurred due to home birth. Thus way the information before this year, 2016 cannot be the representative of the current strategy because the information before the strategy might be occurred due the attention was not given to them to reduce the problem but currently, from 2016 to date full attention is being given and follow up also along the strategy. Thus, the aim of the meta-analysis is to identify the prevalence and factors related to the problem during this focused strategy to prevent homebirth preference which leads to maternal mortality. If these two were mixed, It would be difficult to be representative information with current policy of the country. |
| 4 | Please revise the discussion section | Revised. |
|  | **Reviewer #1** | **Authors’ response** |
| 1 | **Abstract:** |  |
|  | **Background:**  What is the problem? Is it homebirth itself or not being attended by skilled birth attendant? | In Ethiopia, homebirth is being attended by unskilled birth attendant; this can be the reason for many complications, maternal and child mortality. |
|  | **Method** | Method part is revised thoroughly |
|  | **Conclusion** | Improving the knowledge of pregnant women about the benefit of health facility delivery and obstetric danger signs is necessary to decrease the prevalence of homebirth preference; for these can reduce negative outcomes occurred during delivery. |
| 2 | **Background** | |
|  | Regarding the information raised in background | Background is summarized, improved and modified (indicated with track change) |
|  | **Methods** | |
|  | PROSPERO registration number | Still on process |
|  | PRISMA guideline for result writing | Citation 11 |
|  | PICO | A research question used to conduct this systematic review and meta-analysis.  A research question of this review was developed on PICO principle ( pregnant mothers, factors, condition/setting/ Ethiopia, outcome/home birth) |
|  | Data bases | Google Scholar, Medline/Pub Med, Cochrane library, the Web of Science, Hinari, Science Direct, ProQuest, African Journals Online |
|  | Non data bases | online university repositories (University of Gondar, Addis Ababa, Jimma and Haramaya University)  From these online searching (repositories) no researches articles, other than those searched from data bases are found. |
|  | How do authors make sure search terms are not missed? | Before developing search term authors care full read and search for deferent terms with the outcome and factors might be published with the problem under the study |
|  | **Eligibility criteria** | In this systematic review and meta-analysis, we included:-  • All studies that were conducted on homebirth preference and/or associated factors among pregnant women in Ethiopia.  • all types of articles that were published in the form of journal articles  • Master’s theses and dissertations in the English language.  • The restriction was made for the date of publication, articles published before 2016 were excluded because the Ethiopian strategies to overcome the problem of homebirth were critically started since 2016 as transformation strategies and policy. |
|  | Why studies before 2016 were excluded? | The reason is that the research articles published before 2016 were not representative of the current strategy/policy of the country. This means the reality of the country, the current strategy which is from 2016 is the focused strategy to reduce home birth preference to reduce maternal mortality occurred due to home birth. Thus way the information before this year, 2016 cannot be the representative of the current strategy because the information before the strategy might be occurred due the attention was not given to them to reduce the problem but currently, from 2016 to date full attention is being given and follow up also along the strategy. Thus, the aim of the meta-analysis is to identify the prevalence and factors related to the problem during this focused strategy to prevent homebirth preference which leads to maternal mortality. If these two were mixed, It would be difficult to be representative information with current policy of the country. |
|  | Data extraction and quality appraisal | Score of quality (table 1) and **contents** of extraction check lists are explained in manuscripts, study setting is there in table 1 also |
|  | Meta-regression | Was done for quantitative variables(year of publication and sample size) |
|  | sensitivity | Done to see outliers |
|  | What aspects of variations should you consider to bring down this high heterogeneity in addition to sample size and publication year?  Publication bias | No heterogeneity was detected using meta regression table 2 |
|  | The visual examination of the funnel plot presented symmetrically which is an indicator for the absence of publication bias (Fig. 5)” this funnel plot is not symmetry | Egger and begger test shows no publication bias (not significant) as well as the funnel plot is somewhat symmetric even though it is subjective. |
|  | **Reviewer #2** | **Response** |
| **Abstract** | Study setting | Google Scholar, Medline, Pub Med, Cochrane library, and the Web of Science search engines were used to identify research articles for this systematic review and meta-analysis from 20th August 2022 to 6th November 2022, Ethiopia |
|  | Background | Modified |
|  | PICO | A research question used to conduct this systematic review and meta-analysis.  A research question of this review was developed on PICO principle ( pregnant mothers, factors, condition/setting/ Ethiopia, outcome/home birth) |
|  | Classify it as inclusion and exclusion criteria. Based on your PICO guideline. | In this systematic review and meta-analysis, we included:-  • All studies that were conducted on homebirth preference and/or associated factors among pregnant women in Ethiopia.  • All types of articles that were published in the form of journal articles  • Master’s theses and dissertations in the English language.  • The restriction was made for the date of publication, articles published before 2016 were excluded because the Ethiopian strategies to overcome the problem of homebirth were critically started since 2016 as transformation strategies and policy. |
|  | What was the reason for not including studies done before 2016? | The reason is that the research articles published before 2016 were not representative of the current strategy/policy of the country. This means the reality of the country, the current strategy which is from 2016 is the focused strategy to reduce home birth preference to reduce maternal mortality occurred due to home birth. Thus way the information before this year, 2016 cannot be the representative of the current strategy because the information before the strategy might be occurred due the attention was not given to them to reduce the problem but currently, from 2016 to date full attention is being given and follow up also along the strategy. Thus, the aim of the meta-analysis is to identify the prevalence and factors related to the problem during this focused strategy to prevent homebirth preference which leads to maternal mortality. If these two were mixed, It would be difficult to be representative information with current policy of the country. |
