# Supplementary material for: Prevalence of homebirth preference and associated factors among pregnant women in Ethiopia: Systematic review and meta-analysis
Source: PLoS One. 2023 Nov 15;18(11):e0291394. doi: 10.1371/journal.pone.0291394 (PMC10651027; doi:10.1371/journal.pone.0291394)
Supplement: Attachment — Submitted filename: Comment.docx [file pone.0291394.s006.docx]

Comment

Thank you for giving me the opportunity to review the manuscript titled ‘PREVALENCE OF HOMEBIRTH PREFERENCE AND ASSOCIATED FACTORSAMONG PREGNANT WOMEN IN ETHIOPIA: SYSTEMATIC REVIEW AND META-ANALYSIS’

General comment: the manuscript tried to address one of the challenges of maternal and child health in the Ethiopian context and the findings might have some significance for policy implications. By saying this I would try to forward some possible comments and suggestions regarding the manuscript.

- There was another systematic review and meta-analysis on the same topic in 20219 <https://www.ncbi.nlm.nih.gov/pmc/articles/PMC8042927/> ) by using 40 studies which had far better strength to estimate the pooled estimate than the current one. So, what are the new findings and methodologies added to the current study?
- I think there were also some issues regarding the proper use of search engines. I think it was possible to use include other studies on facility delivery by inverting their outcome of interest( for example a study that showed a facility delivery of 71% means that a home delivery of 29%). In addition, almost all of the studies were confined to three regions but there were plenty of studies conducted throughout every corner of the country.
- I also doubt the pooled prevalence. A recent and similar study in 2021 showed 66% whereas the current one showed 39.62%. what could be the possible justification behind these discrepancies?
- The majority of the statements throughout the manuscript were vague to understand and need paraphrasing. For instance, in the methodology section ‘Published studies were included in the study, but unpublished studies were not found.’ What does it mean? in another part, it was mentioned that the authors were using grey literature from various repositories???
- The authors failed to mention the search strategy used to retrieve relevant articles from each electronic database. For instance, what were the Medical Subject Heading (MeSH) and keywords used for PubMed, google scholar…
- The result lacks subgroup analysis by region, publication year, and any other significant attributes. This shows that there is a significant homogeneity in the prevalence of home birth across the country which is unlikely and this might be due to an erroneous way of searching and inclusion of the articles.
- The figures were not eye-catching for the readers and try to modify them
- The figures for the exposure variables were a little bit confusing ‘for example the right side for formal education and the left side for no formal education??? It's a new way of data presentation in the forest plot for me. Once you select the odds ratios that deal with formal education, you can indicate it by interpreting the result in the statement form.
- Reporting an excess number of variables as predictors with merely 7 studies by itself shows the spurious type of relations.
- Discussion section
- To put a strong public health implication, it would be better to compare the findings from the nationally representative data like EDHS 2016 04 mini EDHS 2019...
- Once, the authors tried to address the above-mentioned basic concerns, the manuscript may have the chance to be published in journal

Thank you!
