# Supplementary material for: Prevalence of homebirth preference and associated factors among pregnant women in Ethiopia: Systematic review and meta-analysis
Source: PLoS One. 2023 Nov 15;18(11):e0291394. doi: 10.1371/journal.pone.0291394 (PMC10651027; doi:10.1371/journal.pone.0291394)
Supplement: Attachment — Submitted filename: Response to reviewers and editor.docx [file pone.0291394.s008.docx]

**Responses to Editor and Reviewers**

First of all, we would like to thank the Editor and Reviewers of this manuscript for giving us such constructive comments and questions to enrich this manuscript.

| **S/N** | **Questions/comments** | **Authors’ response** |
| --- | --- | --- |
|  | **General** |  |
|  | **Language and figures** | Clearly modified |
|  | **Reviewer #1** | **Authors’ response** |
| 1 | The language of the manuscript needs to be improved for publication. | Language has been revised |
|  | **Reviewer #3** | **Authors’ response** |
| 1 | Other meta-analysis? | Home birth and preference of home birth is not the title of similar issue. Homebirth preference is the intention/plan to give birth outside health facilities with the help of unskilled birth attendants.  While home birth is the action which indicates giving birth at home. Intention to give birth at home is not mean giving birth at home but their interest for their coming delivery time but they may or may not give birth at home and it can be one of the factors those causes home birth but not limited to it.  Therefore home birth is not preference of homebirth. |
|  | **language** | revised |
|  | **Searching strategy** | It was already described clearly in manuscript |
|  | **Subgroup analysis** | Analyzed separately in the manuscript |
| 2 |  | |
|  | Language and pictures | revised |
