# Supplementary material for: Prevalence of homebirth preference and associated factors among pregnant women in Ethiopia: Systematic review and meta-analysis
Source: PLoS One. 2023 Nov 15;18(11):e0291394. doi: 10.1371/journal.pone.0291394 (PMC10651027; doi:10.1371/journal.pone.0291394)
Supplement: Attachment — Submitted filename: Response to reviewers and editor.docx [file pone.0291394.s009.docx]

**Responses to Editor**

First of all, we would like to thank the Editor of this manuscript for giving us such constructive comments and questions to enrich this manuscript.

| **S/N** | **Questions/comments** | **Authors’ response** |
| --- | --- | --- |
|  | **comments** |  |
| 1 | Title. | Revised |
|  | **abstract** | **Revised** |
| 1 | Methods | Revised indicated by track change within manuscript |
|  | **Result and conclusion** | Revised indicated by track change within manuscript |
